# Uncovering the Therapeutic Potential of Lithium Chloride in Type 2 Diabetic Cardiomyopathy: Targeting Tau Hyperphosphorylation and TGF-β Signaling via GSK-3β Inhibition

**DOI:** 10.3390/pharmaceutics16070955

**Published:** 2024-07-19

**Authors:** Layal Abou Assi, Sahar Alkhansa, Rachel Njeim, Jaafar Ismail, Mikel Madi, Hilda E. Ghadieh, Sarah Al Moussawi, Tanya S. Azar, Maurice Ayoub, William S. Azar, Sarah Hamade, Rashad Nawfal, Nina-Rossa Haddad, Frederic Harb, Wissam Faour, Mahmoud I. Khalil, Assaad A. Eid

**Affiliations:** 1 Department of Biological Sciences, Faculty of Science, Beirut Arab University, Beirut 1107-2020, Lebanon; lka061@student.bau.edu.lb (L.A.A.);; 2 Department of Anatomy, Cell Biology, and Physiological Sciences, Faculty of Medicine, American University of Beirut, Beirut 1107-2020, Lebanon; sma86@mail.aub.edu (S.A.); rmn32@mail.aub.edu (R.N.); jmi05@mail.aub.edu (J.I.); mtm13@mail.aub.edu (M.M.); hilda.ghadieh@balamand.edu.lb (H.E.G.); sm325@aub.edu.lb (S.A.M.); tsa25@mail.aub.edu (T.S.A.); ayoubmaurice123@gmail.com (M.A.); wsa11@georgetown.edu (W.S.A.); swh07@mail.aub.edu (S.H.); rn107@aub.edu.lb (R.N.); 3 AUB Diabetes, American University of Beirut Medical Center, Beirut 1107-2020, Lebanon; nina.haddad07@gmail.com (N.-R.H.); frederic.harb@balamand.edu.lb (F.H.); 4 Department of Biomedical Sciences, Faculty of Medicine, and Medical Sciences, University of Balamand, Tripoli 1300, Lebanon; 5 Faculty of Medicine, Lebanese University, Beirut 1107-2020, Lebanon; 6 Gilbert and Rose-Marie Chagoury School of Medicine, Lebanese American University, Beirut 1107-2020, Lebanon; wissam.faour@lau.edu.lb; 7 Molecular Biology Unit, Department of Zoology, Faculty of Science, Alexandria University, Alexandria 21526, Egypt

**Keywords:** diabetic cardiomyopathy, TGF-β, tau hyperphosphorylation, lithium chloride

## Abstract

Diabetic cardiomyopathy (DCM) is a major complication of type 2 diabetes mellitus (T2DM) that leads to significant morbidity and mortality. The alteration in the signaling mechanism in diabetes leading to cardiomyopathy remains unclear. The purpose of this study is to investigate the role of tauopathy in myocardial dysfunction observed in T2DM. In that regard, diabetic Sprague Dawley rats were treated with intraperitoneal injections of lithium chloride (LiCl), inhibiting tau phosphorylation. Cardiac function was evaluated, and molecular markers of myocardial fibrosis and the TGF-β signaling were analyzed. T2DM rats exhibited a decline in ejection fraction and fractional shortening that revealed cardiac function abnormalities and increased myocardial fibrosis. These changes were associated with tau hyperphosphorylation. Treating diabetic rats with LiCl attenuated cardiac fibrosis and improved myocardial function. Inhibition of GSK-3β leads to the suppression of tau phosphorylation, which is associated with a decrease in TGF-β expression and regulation of the pro-inflammatory markers, suggesting that tau hyperphosphorylation is parallelly associated with fibrosis and inflammation in the diabetic heart. Our findings provide evidence of a possible role of tau hyperphosphorylation in the pathogenesis of DCM through the activation of TGF-β and by inducing inflammation. Targeting the inhibition of tau phosphorylation may offer novel therapeutic approaches to reduce DCM burden in T2DM patients.

## 1. Introduction

Diabetes mellitus, a major global metabolic disorder, is on the rise at an alarming rate. Diabetic cardiomyopathy (DCM) is defined as the most common chronic diabetic complication leading to heart failure (HF) in the absence of hypertension and coronary artery disease through a variety of mechanisms [1]. These mechanisms comprise disease-specific myocardial structural, functional, and metabolic abnormalities, reactivation of the “fetal gene program”, impaired cardiac contractility, cardiomyocyte hypertrophy, cellular apoptosis, and interstitial myocardial fibrosis. The heart adapts to diabetic-induced environmental changes through the rearrangement of extracellular matrix (ECM) proteins, including imbalanced collagen deposition, and cardiomyocyte hypertrophy [2]. Ventricular compliance is reduced, leading to diastolic dysfunction, which eventually progresses to contractile dysfunction [3]. Furthermore, the shifts in the myosin heavy chain composition (β/α-MyHC ratio) and endothelial-to-mesenchymal transition, where the endothelial cells lose endothelial markers and gain mesenchymal features such as α-smooth muscle actin (α-SMA), are important cellular phenotype shifts that aggravate cardiac fibrosis and are observed in DCM [4,5]. Therefore, studies are still warranted to understand the mechanistic pathways behind these cardiac phenotypic alterations.

Activated transforming growth factor beta (TGF-β) leads to cardiac fibrosis and mediates endothelial-to-mesenchymal transition which in turn induces DCM through the SMAD-dependent and independent pathways [6]. Yet, the activating mechanisms of TGF-β still need to be elucidated. Herein, we identify a potential molecular pathway that can activate TGF-β leading to cardiac fibrosis, which induces the pathological changes of DCM. 

Tau, a highly soluble, natively unfolded protein, is a “microtubule-stabilizing protein” responsible for intracellular transport, axonal morphology, and cell physiology [7]. Tau is highly expressed in various tissues besides the nervous system, such as the heart, kidneys, skeletal muscle, testis, and lungs. It is slightly expressed in the liver, adrenal gland, and stomach [8]. Tau protein functions predominantly in a phosphorylation-dependent manner mainly through protein kinases, including glycogen synthase kinase-3β (GSK-3β) [9]. GSK-3β is a proline-directed tau kinase and a serine/threonine protein kinase responsible for tau phosphorylation at S214 [10]. Hyperphosphorylated tau tends to dissociate from the cytoskeleton and aggregate, which results in decreased affinity for microtubules [11]. Tauopathies differ based on the hyperphosphorylation states among and within disorders [7]. It is important to note that excess intracellular glucose has been shown to activate tau protein kinases and induce its hyperphosphorylation [12]. However, so far there are limited studies that reveal any change that may occur to tau in any cardiac disease, including DCM.

To our knowledge, our study stands as a pioneering effort, representing a comprehensive exploration of the mechanistic pathway that potentially underlines lithium’s actions. Our research delves into its impact on the regulation of inflammation associated with diabetes, as well as its involvement in diabetic complications. Furthermore, we emphasize its pivotal role in modulating cardiac fibrosis induced by TGF-β. This novel and innovative aspect distinguishes our paper from previous works in the field.

## 2. Materials and Methods

### 2.1. Animal Models

All animal procedures were conducted in accordance with the Institutional Animal Care and Use Committee (IACUC) of the American University of Beirut (Beirut, Lebanon). One-month-old male Sprague Dawley rats, weighing between 100 g and 200 g, were divided into 4 groups, each group comprising 5 rats (n = 5). The high-fat diet (HFD) and streptozotocin (STZ) model was employed for inducing diabetes. This widely studied and well-established HFD/STZ model is commonly used to simulate type 2 diabetes mellitus (T2DM) [13,14]. The model involves a combination of a high-fat diet (HFD) regimen along with low-dose streptozotocin (STZ) treatment. This approach is designed to replicate the natural progression of the disease and mimic metabolic characteristics observed in human type 2 diabetes mellitus (T2DM), such as insulin resistance [15]. Furthermore, this model has been specifically associated with the development of diabetic cardiomyopathy (DCM) following a short-term diet intervention [16]. Three groups of rats were fed an HFD (60% kcal of fat) for eight weeks, followed by a single intraperitoneal injection (I.P.) of a low dose of 30 mg/kg body weight streptozotocin (STZ; Sigma-Aldrich, Darmstadt, Germany) dissolved in sodium citrate buffer (0.1 M, pH 4.5) to induce T2DM [14]. Blood glucose levels were measured afterward (AccuCheck Performa, Roche, Basel, Switzerland) to verify the onset of diabetes. Rats with glucose levels of >250 mg/dL were considered diabetic. The fourth control group was maintained on a normal diet (11% kcal of fat) and treated with sodium citrate buffer as a vehicle. To inhibit tau phosphorylation, we used lithium chloride (LiCl). LiCl treatment has been widely used to prevent tau phosphorylation through GSK-3β inhibition [17,18]. LiCl (Thermo Fisher Scientific, Waltham, MA, USA) was dissolved in normal saline and intraperitoneally injected at a dose of 1  mmol/kg [19].

The three groups of rats fed an HFD were randomly divided as follows: (1) T2DM group treated with the vehicle, (2) T2DM group treated intraperitoneally with 1 mmol/kg body weight of lithium chloride (LiCl) daily, and (3) T2DM group treated intraperitoneally with 1 mmol/kg body weight of LiCl every other day. Treatments with LiCl were initiated at the onset of diabetes and all groups of rats were kept in a temperature-controlled room and on a 12/12 dark/light cycle and had unrestricted access to food and water for 14 weeks. Glucose concentration was monitored weekly in the blood collected from the rats using a LifeScan One Touch glucometer (AccuCheck Performa, Roche, Basel, Switzerland). At the end of the 14 weeks of treatment, cardiac function was evaluated. After echocardiography, rats were euthanized and the left ventricles (LVs) of the hearts were collected, weighed, and stored at −80 °C for biochemical assays or fixed with 4% formaldehyde for histological analysis.

### 2.2. Echocardiography

Echocardiography was performed in order to evaluate the cardiac function in the studied animals as previously described [4,20]. Rats’ echocardiography was performed with a linear 40-8 MHz transducer (model L40-8/12, Ultrasonix Medical Corporation, Richmond, BC, Canada) connected to a high-performance ultrasound system (Ultrasonix Medical Corporation). Rats were anesthetized and positioned horizontally on a controlling heating pad to maintain their normal body temperature (37 °C) and their anterior chest wall was shaved to place the transducer. Two-dimensional echocardiography images were obtained using M-mode, in the parasternal short- and long-axis views. LV end-diastolic diameter (LVEDD), LV end-systolic diameter (LVESD), LV end-diastolic volume (LVEDV), LV end-systolic volume (LVESV), and LV mass (LVM) were measured. LV fractional shortening (FS%) and LV ejection fraction percentages (EF%) were calculated as follows: [(LVEDD − LVESD)/LVEDD] × 100 (%) and [(LVEDV − LVESV)/LVEDV] × 100 (%), respectively. Measurements at each time point were averaged based on six different cardiac cycles [4,20].

### 2.3. ELISA Measurement in Serum Samples

Blood was collected on the day of sacrifice. Blood was centrifuged at 3000 rpm for 10 min at 4 °C to separate the plasma from the red blood cells. Insulin levels were measured using a Rat INS (Insulin) ELISA Kit (Catalogue No ER1113; Fine test, Wuhan, China). Free fatty acid (FFA) levels were measured using a Free Fatty Acid Assay Kit (ab65341; Abcam, Cambridge, UK). Triglyceride (TG) levels were measured using a Triglyceride Assay Kit (ab65336; Abcam, Cambridge, UK). Plasma levels of N-terminal pro-brain natriuretic peptide (NT-proBNP) (CSB-E08752r) and cardiac troponin T (cTnT) (CSB-E16443r) were determined using an enzyme-linked immunosorbent assay (ELISA) method (Cusabio, Houston, TX, USA), according to the manufacturer’s protocol.

### 2.4. LV Histology

Masson’s trichrome and periodic acid–Schiff (PAS) stains were applied on 4 µm sections of the LV to obtain a clear histomorphological image of cardiac tissue remodeling. Masson’s trichrome staining is used to assess collagen deposition. Periodic acid–Schiff staining was used to determine the volume density of glycogen storage (magenta color). Results were then further investigated under a light microscope (10×) (Olympus, CX41, Tokyo, Japan) and analysis was performed using ImageJ software (1.53 e, National Institutes of Health, Bethesda, MD, USA) as previously described [4].

### 2.5. Immunohistochemistry Analysis

β-MyHC and TGF-β1 were detected on 4 µm thick slices following deparaffinization and antigen retrieval as previously described [4,6]. Sections were incubated with the following primary antibodies: mouse anti-skeletal muscle myosin (S58) (1:50; catalog No. sc-32733, Santa Cruz Biotechnology, Dallas, TX, USA) and rabbit anti-TGF-β1 (1:100; catalog No. G122A, Promega, Madison, WI, USA), which were detected using Novolink™ Polymer Detection System kit (Leica Biosystems, Buffalo Grove, IL, USA), according to the manufacturer’s protocol. Protein expression was assessed by estimating the brown-colored area and the results were investigated under a light microscope (10×) (Olympus, CX41, Tokyo, Japan) and represented by the percentage of area fraction compared to controls. Image analysis was performed using Image GraphPad Prism (9.3.1 software, San Diego, CA, USA).

### 2.6. Western Blot Analysis

Homogenates from LV were lysed in 250 µL of radioimmunoprecipitation assay buffer (0.1% sodium dodecyl sulfate, 0.5% sodium deoxycholate, 300 mM NaCl, 100 mM Tris-HCl pH 8, 1% NP-40, Protease Inhibitor Cocktail, Phosphatase Inhibitor Cocktail, and 1 mM phenylmethanesulfonylfluoride (PMSF)) using a homogenizer. Homogenates were placed on a rotator overnight and centrifuged at 13,200 RPM for 30 min at 4 °C to obtain a supernatant. Protein in the supernatants was measured using a Bio-Rad protein assay. For immunoblotting, proteins (60 µg) were separated by 8% SDS-PAGE and transferred to polyvinylidene difluoride membranes. The membranes were blocked with 5% bovine serum albumin (BSA) in Tris-buffered saline and then incubated with primary antibodies. The antibodies used include the rabbit polyclonal anti-tau (phospho Ser214) antibody (dilution 1:250; catalog No. ab10891; Abcam, Cambridge, UK), rabbit polyclonal anti-tau antibody (dilution 1:300; catalog No. bs-0157R; Bioss Antibodies, Beijing, China), rabbit polyclonal anti-GSK-3-beta (phospho Ser9) (dilution 1:1000, catalog No. 9322; cell signaling, Danvers, MA, USA), rabbit polyclonal anti-GSK-3-beta (dilution 1:1000, catalog No. 9332; cell signaling, Danvers, MA, USA), goat polyclonal anti-α-MyHC (dilution 1:500, sc-168676, Santa Cruz Biotechnology, Dallas, TX, USA), rabbit polyclonal anti-alpha α-SMA antibody (dilution 1:200; catalog No. ab5694; Abcam, Cambridge, UK), rabbit polyclonal anti-smad3 (phospho Ser423/425) (dilution 1:1000, catalog No. 9520; cell signaling, Danvers, MA, USA), rabbit polyclonal anti-smad3 (dilution 1:1000, catalog No 9523; cell signaling, Danvers, MA, USA), and mouse monoclonal anti-HSC70 (B-6) antibody (1:500; catalog No. sc-7298; Santa Cruz Biotechnology, Inc., Dallas, TX, USA). In our study, HSC70 was selected as a loading control because it is consistently expressed in different tissues and remains stable under various conditions. This ensures accurate normalization and quantification in our protein analyses [4,21,22]. The primary antibodies were detected using horseradish peroxidase-conjugated IgG (dilution 1:5000, 170-5046, Bio-Rad Laboratories, Hercules, CA, USA and ab97100, Abcam, Cambridge, UK). Bands were visualized by enhanced chemiluminescence. Densitometric analysis was performed using ImageJ software (1.53 e, National Institutes of Health, Bethesda, MD, USA) [23,24].

### 2.7. PCR Analysis

mRNA was analyzed by real-time RT-PCR using the ΔΔCt method. Total RNA was extracted from LV using TRIZOL reagent (Sigma-Aldrich, St. Louis, MO, USA) and converted into cDNA using the Revert First Strand cDNA Synthesis Kit (Qiagen, Hilden, Germany) according to the manufacturer’s protocol. mRNA expression was quantified using the CFX384 Touch (Bio-Rad, Hercules, CA, USA) with SYBR Green dye and predesigned rat RT^2^-quantitative PCR primers. Fibronectin: forward 5′-GATGGAATCCGGGAGCTTTT-3′ and reverse 5′-TGCAAGGCAACCACACTGAC-3′; collagen I: forward 5′-ATCAGCCCAAACCCCAAGGAGA-3′ and reverse 5′-CGCAGGAAGGTCAGCTGGATAG-3′; α-MyHC: forward 5’-AGCCTCTCATCTCGCATCTC-3’ and reverse 5’-GGACCACCCATCTCACTTT-3′; β-MyHC: forward 5′-ACCGCTGAGACAGAGAATGG-3’ and reverse 5′-GGGTTGGCTTGGATGATTT-3′; α-SMA: forward 5′-TGGCTATTCCTTCGTTACTACTGCT-3′ and reverse 5′-CATCAGGCAACTCGTAACTCTTCTC-3′; TGF-β1: forward 5′-CCCCTGGAAAGGGCTCAACAC-3′ and reverse 5′-TCCAACCCAGGTCCTTCCTAAAGTC-3′; 26s: forward 5′-AGGAGAAACAACGGTCGTGCCAAAA-3′ and reverse 5′-GCGCAAGCAGGTCTGAATCGTG-3′ was used as an internal reference gene.

### 2.8. Statistical Analysis

Data analysis was performed using GraphPad Prism 9 (9.4.1 software, San Diego, CA, USA). Descriptive statistics with frequencies and percentages were used for categorical variables and mean ± standard deviation (SD) or mean ± standard error of the mean (SEM) for continuous variables. The Student’s *t*-test was used to compare two means whereas the ANOVA test was used when the comparison involved three or more groups, followed by Fisher’s LSD or Tukey’s post hoc tests. Our statistical approach was chosen after conducting a Shapiro–Wilk test to assess the normality of our data. The results of the normality test indicated that all the groups in each study design passed the normality test, allowing us to use a parametric test for the statistical analysis. A value of *p* < 0.05 was considered significant. 

## 3. Results

### 3.1. Metabolic Profile Alterations in T2DM Rats

After 14 weeks of T2DM onset and a 22-week period of HFD, the untreated T2DM group displayed a significant increase in body weight (mean ± SEM) compared to controls. However, daily treatment with 1 mmol/kg LiCl for 14 weeks significantly reduced the body weight of T2DM rats compared to untreated T2DM rats, while treatment with LiCl every other day did not show the same effect. Table 1 provides a summary of the observed changes in body weight.

The blood glucose levels (mg/dL) of untreated T2DM rats were significantly higher compared to control rats fed standard chow. While T2DM rats treated with 1 mmol/kg LiCl daily and every other day did not reverse the increase in blood glucose levels, there was a slight decrease in blood glucose concentrations observed in rats treated daily with LiCl, which could be explained by the decrease in body weight. Moreover, insulin levels exhibited a significant increase in T2DM control rats compared to the control group, indicating the development of insulin resistance. Interestingly, daily or every other day treatment with LiCl led to a decrease in insulin levels, although they remained significantly higher than those in the control rats. Additionally, we assessed concentrations of triglycerides (TG) and free fatty acids (FFAs) to investigate the presence of hyperlipidemia, a crucial parameter in T2DM. Both FFA and TG showed a significant increase in T2DM, and upon treatment with LiCl daily or every other day, there was a statistically significant decrease. These findings suggest that treatment with lithium chloride significantly mitigated hyperlipidemia (Table 1).

To assess hypertrophy, the HW/TL ratio (the ratio of heart weight (g) to tibia length (cm), (g/cm)) was calculated. The HW/TL ratio significantly increased with diabetes, indicating hypertrophy. However, after treatment with LiCl, the HW/TL ratio significantly decreased compared to untreated T2DM rats, suggesting that LiCl treatment improved the structural characteristics of T2DM rats.

### 3.2. T2DM Induces Hyperphosphorylation of Tau Protein in the Rats’ LV

**Figure 1:**
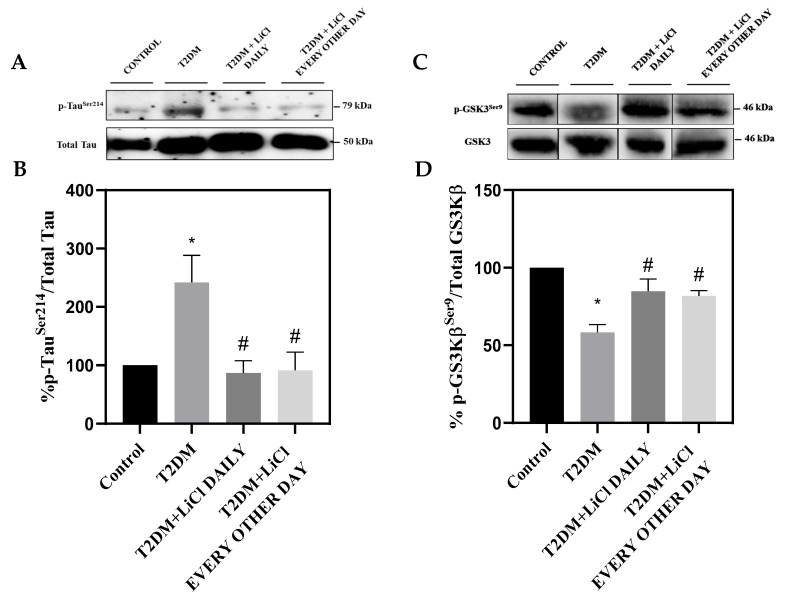
Effect of LiCl treatment on tau phosphorylation in the LV of T2DM rats. (**A**) Representative Western blot of p-Tau^Ser214^ and total tau proteins. (**B**) Bar graph showing the quantification of p-Tau^Ser214^ to total tau protein in LV tissue samples from control rats, rats with T2DM, and T2DM rats treated with LiCl daily or every other day. (**C**) Representative Western blot of GSK-3β^Ser9^ and total GSK-3β proteins. (**D**) Bar graph showing the quantification of GSK-3β^Ser9^ to total GSK-3β protein in LV tissue samples from all groups. The data are presented as means ± SD with n = 5/group. Statistical significance is denoted by * *p* < 0.05 vs. control rats, # *p* < 0.05 vs. diabetic rats. Full pictures of the Western blots and the densitometry ratio are presented in Figure S1 and Table S1.

To investigate the impact of diabetes on tau phosphorylation, we analyzed the expression of phosphorylated tau protein in the left ventricular lysates of the four different groups of rats. Our Western blot analysis revealed a dominant 79 kDa band of phospho-Tau^Ser214^, confirming hyperphosphorylation of tau in the LVs of untreated T2DM rats when compared to controls (Figure 1A,B). Interestingly, treatment with LiCl significantly attenuated tau phosphorylation in the LV of T2DM rats when administered either daily or every other day by increasing GSK-3β phosphorylation at its inhibitory site Ser9 (Figure 1A–D). These observations suggest that T2DM can lead to hyperphosphorylation of tau protein in the heart, which may contribute to the development of cardiac complications.

### 3.3. Tau Hyperphosphorylation Inhibition by LiCl Is Associated with Attenuation of Cardiac Dysfunction and Structural Changes

To study the link between tau hyperphosphorylation and cardiac structure and hemodynamics, echocardiography was performed in the four different rat groups. Cardiac hypertrophy, which is commonly seen in DCM, was evident by the significant increase in the LVM-to-tibia-length ratio in the untreated T2DM group when compared to the control group (Table 2). Interestingly, the increase in LVM-to-tibia-length ratio was significantly attenuated in the T2DM groups treated with LiCl daily or every other day compared to untreated diabetic rats (Table 2). Additionally, the echocardiography measurements revealed an increase in systolic thicknesses (ESDs) (mm) of the interventricular septum and the posterior wall, as well as the end-systolic volumes (ESVs) (mL), in the untreated T2DM group when compared to the control group (*p* < 0.05). These changes may be indicative of systolic dysfunction, which is commonly observed in DCM. However, treatment with LiCl daily or every other day resulted in a decrease in these measurements (Table 2).

**Figure 2:**
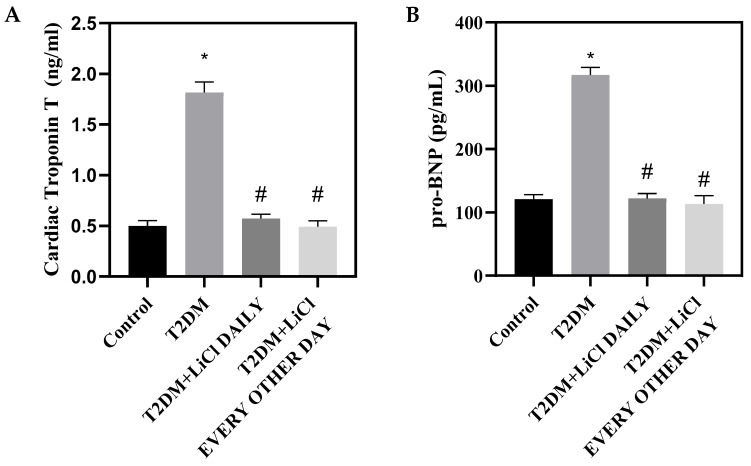
LiCl treatment attenuates cardiac injury. (**A**) Bar graph showing the levels of cTnT and (**B**) bar graph showing the levels of NT-proBNP in the plasma from control rats, rats with T2DM, and T2DM rats treated with LiCl daily or every other day. The data are presented as means ± SD with n = 5/group. Statistical significance is denoted by * *p* < 0.05 vs. control rats, # *p* < 0.05 vs. diabetic rats.

We further measured the plasma levels of cTnT and NT-proBNP, widely used biomarkers for cardiac injury. We observed elevated levels of both cTnT and NT-proBNP in the plasma samples of diabetic rats, suggestive of cardiac injury. However, a notable reduction in these markers was seen when the rats were administered LiCl either daily or every other day, proposing a beneficial effect of decreasing tau hyperphosphorylation on mitigating cardiac injury (Figure 2A,B). Taken together, these data suggest that the attenuation of tau hyperphosphorylation with LiCl treatment has a positive effect on cardiac structure and hemodynamics, potentially indicating a protective effect against DCM.

### 3.4. Cardiac Fibrosis Is Correlated to Tau Hyperphosphorylation in T2DM and Is Attenuated by LiCl Treatment

**Figure 3:**
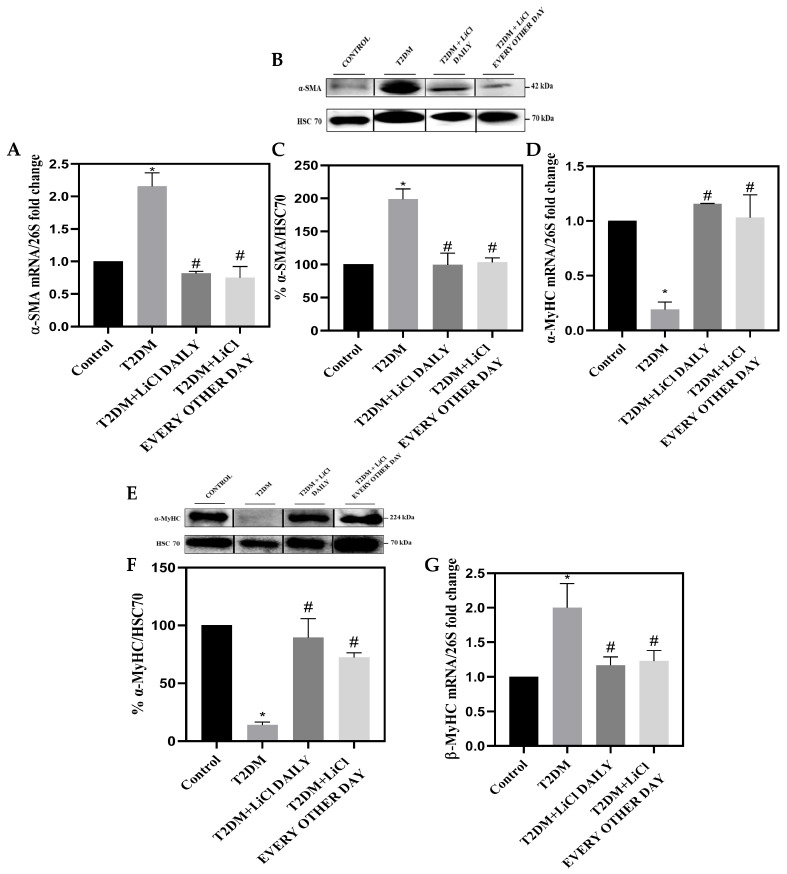

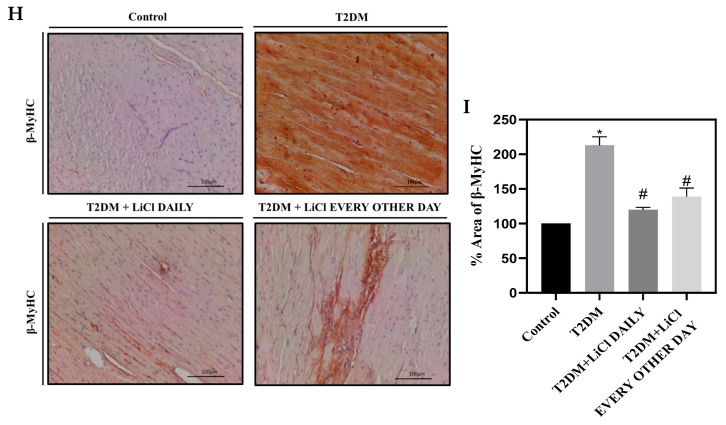
Myocardial injuries in the LV tissue samples from control, T2DM, and lithium-treated T2DM rats. (**A**) Bar graph showing mRNA levels of α-SMA relative to 26S. (**B**) Representative Western blot analysis of α-SMA and Hsc70 protein expression in heart LV tissue samples from control rats, rats with T2DM, and T2DM rats treated with LiCl daily or every other day. (**C**) Bar graph showing the quantification of the α-SMA Western blot. (**D**) Bar graph showing mRNA levels of α-MyHC relative to 26S. (**E**) Representative Western blot analysis of α-MyHC and Hsc70 protein expression in heart LV tissue samples from the studied groups. (**F**) Bar graph showing the quantification of the α-MyHC Western blot. (**G**) Bar graph showing mRNA levels of β-MyHC relative to 26S. (**H**) Representative histopathological sections of the LV stained for β-MyHC (brown color) from the studied groups. Scale bar = 100 µm. (**I**) Bar graph showing the quantification of β-MyHC immunoperoxidase staining. The data are presented as means ± SD with n = 5/group. Statistical significance is denoted by * *p* < 0.05 vs. control rats, # *p* < 0.05 vs. diabetic rats. The Western blot gels represent cropped bands from the same gel where more than one sample is represented and are presented for aesthetic purposes. Full pictures of the Western blots and the densitometry ratio are presented in Figure S2 and Table S1.

**Figure 4:**
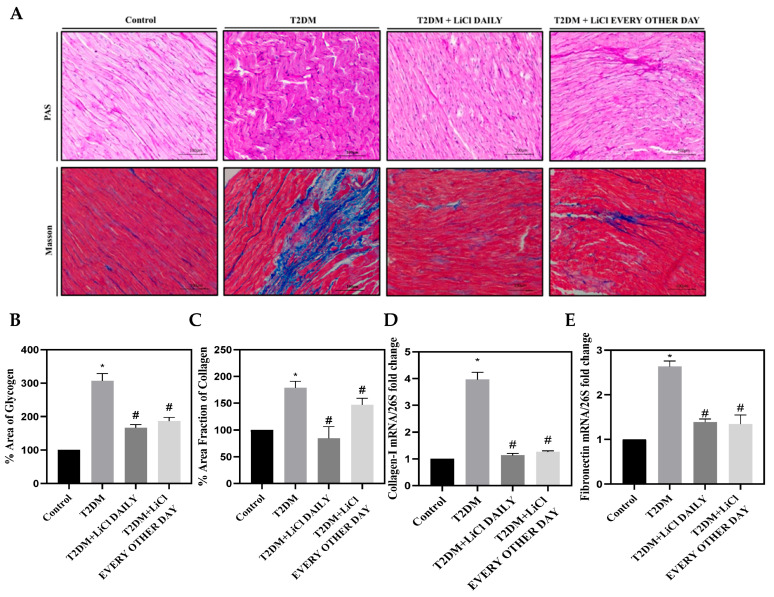
Interstitial cardiac fibrosis. (**A**) Representative micrograph (10× objective) of periodic acid–Schiff (PAS) staining of LV sections showing glycogen extent and distribution in highly fibrotic areas (magenta color) and Masson’s trichrome staining of LV sections showing collagen deposition in highly fibrotic areas (blue-stained ECM). Scale bar = 100 µm. (**B**) Bar graph showing quantification of glycogen deposition in highly fibrotic areas. (**C**) Bar graph showing quantification of collagen deposition in highly fibrotic areas in the LV sections from the studied groups. (**D**) Bar graph showing mRNA levels of collagen I relative to *26S*. (**E**) Bar graph showing mRNA levels of fibronectin relative to *26S*. Data are presented as means ± SD with n = 5/group. Statistical significance is denoted by * *p* < 0.05 vs. control rats, # *p* < 0.05 vs. diabetic rats.

Next, we investigated the relationship between tau hyperphosphorylation and cardiac fibrosis in T2DM. We examined changes in both mRNA and protein expression levels of α-MyHC, β-MyHC, and α-SMA in control, T2DM, and T2DM rats treated with 1 mmol/kg LiCl daily or every other day. We also assessed interstitial cardiac fibrosis using Masson’s trichrome staining and periodic acid–Schiff staining. Our results show that untreated T2DM rats had increased levels of α-SMA and β-MyHC compared to control rats and had decreased levels of α-MyHC (Figure 3A–I). Inhibition of tau hyperphosphorylation through daily and every other day treatments with LiCl was associated with a reduction in both α-SMA and β-MyHC levels and an increase in α-MyHC expression (Figure 3A–I). These findings suggest a possible role of tau hyperphosphorylation in cardiac fibrosis and highlight the potential protective effects of LiCl treatment. Furthermore, our study shows that untreated T2DM rats exhibited a significant increase in glycogen deposition and interstitial cardiac fibrosis when compared to control rats (*p* < 0.05), as assessed by PAS and Masson’s trichrome staining (Figure 4A–C), and increased mRNA expression of collagen I and fibronectin (Figure 4D,E). Interestingly, treatment with 1 mmol/kg LiCl daily or every other day significantly reduced glycogen deposition and myocardial fibrosis in T2DM rats (Figure 4A–E).

Taken together, our findings suggest that tau hyperphosphorylation might play a significant role in cardiac fibrosis in T2DM, and LiCl treatment can ameliorate tau-hyperphosphorylation-induced cardiac injury.

### 3.5. Tau Hyperphosphorylation Is Coupled with Cardiac Inflammation in T2DM and Is Attenuated by LiCl Treatment

**Figure 5:**
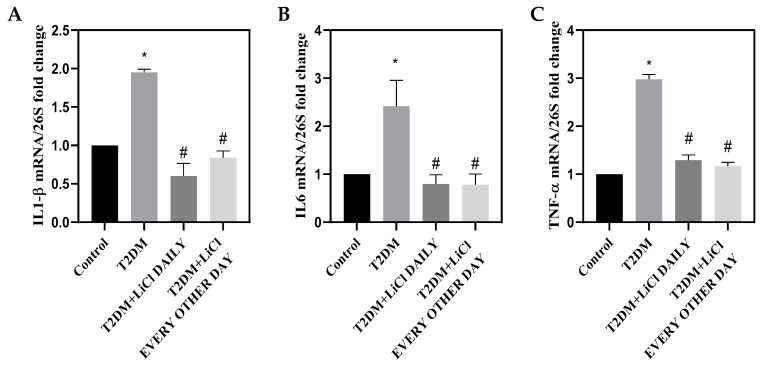
Cardiac Inflammation. (**A**) Bar graph showing mRNA levels of *IL1-β* relative to *26S*. (**B**) Bar graph showing mRNA levels of *IL-6* relative to *26S*. (**C**) Bar graph showing mRNA levels of *TNF-α* relative to *26S*. Data are presented as means ± SD with n = 5/group. Statistical significance is denoted by * *p* < 0.05 vs. control rats, # *p* < 0.05 vs. diabetic rats.

Inflammation plays an important role in the development and progression of DCM, causing pathological changes in cardiac structure and function. Elevated glucose in diabetes triggers an inflammatory response, leading to elevated inflammatory cytokines, oxidative stress, and activation of various inflammatory pathways [25]. Therefore, we examined the association between tau hyperphosphorylation and cardiac inflammation. Our results show that tau hyperphosphorylation is associated with an increase in the mRNA expression of *IL-1β*, *IL-6*, and tumor necrosis factor-α (*TNF-α*) known to play a major role in cardiac remodeling and injury (Figure 5A–C) [26]. Interestingly, treatment with LiCl daily or every other day significantly decreased *IL-1β*, *IL-6*, and *TNF-α* expression (Figure 5A–C). These data suggest that inhibiting tau hyperphosphorylation could disrupt the inflammatory cascade implicated in DCM, thereby reducing cardiac fibrosis, hypertrophy, and dysfunction.

### 3.6. LiCl Treatment Attenuates TGF-β1 Overexpression in the Heart of Type 2 Diabetic Rats

**Figure 6:**
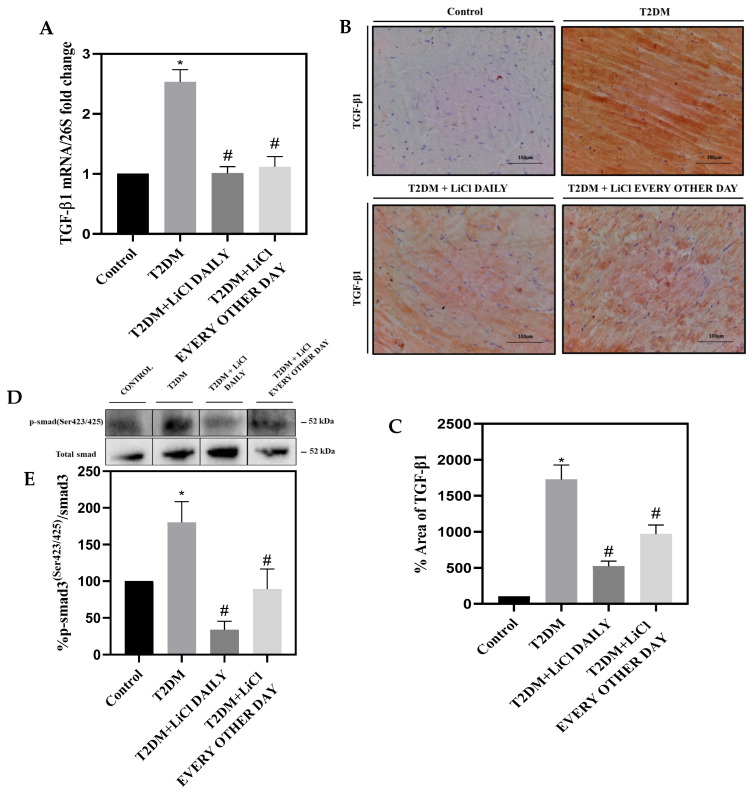
Tau hyperphosphorylation induces cardiac injury by upregulating TGF-β. (**A**) Bar graph showing mRNA levels of *TGF-β1* relative to *26S*. (**B**) Representative micrograph (10× objective) of TGF-β1 immunoperoxidase staining of LV sections, with fibrosis indicated by brown color, from the studied groups. Scale bar = 100 µm. (**C**) Bar graphs showing TGF-β1 quantification. (**D**) Representative Western blot of p-smad3^Ser423/425^ and total smad3 proteins with a (**E**) bar graph showing the quantification of p-smad3^Ser423/425^ to total smad3 protein. Data are presented as means ± SEM with n = 5 per group. Statistical significance is denoted by * *p* < 0.05 vs. control rats, # *p* < 0.05 vs. diabetic rats. Full pictures of the Western blots and the densitometry ratio are presented in Figure S3 and Table S1.

To investigate the possible mechanisms underlying tau-hyperphosphorylation-induced cardiac injury in the context of hyperglycemia, we examined the role of TGF-β1 in the hearts of T2DM rats. TGF-β1 has been implicated in the pathogenesis of cardiac remodeling [6], and its overexpression has been observed in T2DM. Strikingly, treatment with 1 mmol/kg LiCl daily or every other day significantly reduced the expression of both mRNA and protein levels of TGF-β1 in diabetic hearts compared to untreated T2DM rats (Figure 6A–C), suggesting a correlation between tau protein hyperphosphorylation and TGF-β1 activation in the hearts of T2DM rats. Furthermore, our findings demonstrate an increase in smad3 phosphorylation at Ser423/425 in the LV of diabetic rats. Interestingly, this increase was significantly reduced upon treatment with LiCl daily or every other day (Figure 6D,E). This alteration in smad3 phosphorylation, a key downstream effect of TGF-β1 signaling, provides additional evidence of the connection between tau hyperphosphorylation and the TGF-β1 pathway in the development of cardiac remodeling in T2DM.

## 4. Discussion

This study elucidates a potential role of tau hyperphosphorylation in the pathogenesis of DCM through the activation of the TGF-β signaling pathway; this was attributable to the declined cardiac function and overproduction of myocardial fibrosis molecular markers and inflammation. Treatment of T2DM rats with LiCl attenuated cardiac fibrosis and inflammation and improved myocardial function. We have demonstrated that the inhibition of tau protein hyperphosphorylation is associated with a decrease in TGF-β expression induced by DCM.

To better explore the role of tau protein hyperphosphorylation in T2DM we used a therapeutic dose limit of 1 mmol/kg LiCl administered intraperitoneally to HFD-fed STZ-treated rats either daily or every other day for 14 weeks [27]. It is important to note that 1 mmol/kg of LiCl is a pharmacologically tolerated dose that does not cause injury [19], whereas higher doses of LiCl treatment have been linked to causing toxicity, weight loss, and even death [27,28,29]. LiCl treatment has been widely used to prevent tau phosphorylation through GSK-3β inhibition [17] and to mimic insulin-like effects, thus increasing glucose uptake, regulating glucose metabolism [30], and inhibiting myo-inositol-1-monophosphatase [31], 1,6 bisphosphatase, and other enzymes related to glucose metabolism regulation [32]. The observed effect of LiCl may account for the slight decrease in both blood glucose and insulin levels noted after treatment, a reduction similar to that documented in a prior publication [33]. Additionally, we identified significant elevations in both free fatty acids and triglycerides, aligning with the metabolic dysregulation commonly associated with the T2DM disease state. Remarkably, treatment with LiCl substantially mitigated these alterations, signifying a noteworthy reduction in both parameters. This observation aligns with previous studies, further supporting the role of LiCl in ameliorating lipid metabolic dysfunctions [34,35]. It is noteworthy to mention that the protective effect associated with tau inhibition was observed despite the sustained and significant dyslipidemia and insulin resistance.

In this study, T2DM rats exhibited features of DCM, as evidenced by functional and structural alterations in the myocardium, as well as impaired cardiac function. Our findings are supported by previous studies demonstrating that HFD rats exhibit a reduction of FS and an increase in LVM after six weeks of the diet [36]. Additionally, mice fed a high-fat or high-sucrose diet for 16 weeks showed a decrease in echocardiography findings, primarily in EF and FS, leading to impaired systolic function [37].

Interestingly, in our study, daily and every other day treatment with LiCl each restored echocardiography measurements of EF, FS, and LVM parameters. This suggests a potential therapeutic effect of LiCl in DCM.

NT-proBNP and cTnT are important biomarkers in the diagnosis and monitoring of cardiac conditions including diabetic heart disease [38,39]. Elevated proBNP levels may indicate ventricular dysfunction and HF, which are often prevalent in individuals with DCM. Meanwhile, elevated cTnT levels can be an indicator of myocardial damage or stress, which is often undetectable in patients with diabetes due to silent cardiomyopathy. In our T2DM rat model, both NT-proBNP and cTnT were significantly elevated. Notably, inhibition of tau phosphorylation using LiCl was associated with a decrease in both NT-proBNP and cTnT levels, further verifying the effect of tau hyperphosphorylation in inducing cardiac injury.

Moreover, in this study, we found that the myocardial dysfunction observed in our T2DM rats was supported by molecular and histological changes, including an increase in fibrosis and collagen deposition, as well as alterations in the α-SMA and the cardiac fetal gene programming (*α-MyHC* and *β-MyHC*). *β-MyHC*, a characteristic of the entire “fetal gene program”, has been associated with the molecular phenotype changes of the myocardium, and *α-MyHC* has been correlated with systolic dysfunction [4]. Normally, β-MyHC is negligible in adult cardiomyocytes, but overexpression of β-MyHC can act as a compensatory mechanism or reprogramming mechanism of adult cardiomyocytes in response to cardiac pathology and can therefore be considered a biomarker for cardiac hypertrophy [40]. Fetal gene expression has been shown to contribute to contractile dysfunction in diabetic animals [4]. Additionally, α-SMA is typically expressed in differentiated adult cardiomyocytes and is considered a marker for myocardial injury and hypertrophy when upregulated [41]. α-SMA is upregulated following endothelial–mesenchymal transition, and the emergence of endothelial cell-derived fibroblasts, known as myofibroblasts, contributes to cardiac fibrosis [5]. Of interest, in this study, we found that daily, as well as every other day, administration of LiCl attenuated the myocardial injury biomarker expressions observed in T2DM rats. These results suggest that regulating tau phosphorylation might have a significant protective and reversing effect against cardiac hypertrophy and fibrosis reflected by the regulation of β-MyHC, α-MyHC, and α-SMA after treatment.

Furthermore, cardiac fibroblasts are essential for maintaining the structural integrity of the heart, as they are the most abundant cell type in the heart responsible for producing and maintaining the ECM [42]. However, upon cardiac injury, these cells differentiate into myofibroblasts that secrete an abundance of collagen and other proteins, leading to an imbalanced ECM [43]. In diabetic hearts, collagen deposition is reflective of myofibroblast localization to fibrotic areas, highlighting the detrimental effects of uncontrolled hyperglycemia even in relatively early stages of diabetes [44]. Interestingly, our data demonstrate an accumulation of matrix proteins in the hearts of T2DM rats, further underscoring the pathological effects of uncontrolled hyperglycemia on the heart. In addition, our findings suggest a potential association between tau hyperphosphorylation and cardiac pathology, as inhibition of tau protein hyperphosphorylation through treatment with LiCl, independently of correcting glucose levels, improved fibrotic areas in the heart. These results provide evidence for a possible role of the tau protein in the development of DCM and offer exciting prospects for future research and novel therapeutic strategies.

Inflammation is another pathological factor in the etiology of DCM. In T2DM, chronic myocardial inflammation drives morphological and metabolic alterations such as cardiomyocyte apoptosis, impaired calcium management, hypertrophy, and fibrosis which can eventually cause HF [45]. Among the different cytokines, IL-1β, IL-6, and TNF-α have been reported to play a crucial role in cardiac injury [46]. It has been shown that in fibroblast IL-1β, IL-6, and TNF-α inhibit proliferation, reduce matrix synthesis, and enhance MMP activity, while in cardiomyocytes they stimulate apoptosis and hypertrophy [47,48,49,50]. Notably, IL-1β, *IL-6*, and *TNF-α* were all upregulated in the LV of T2DM rats. Importantly, treatment with LiCl was able to significantly downregulate the three cytokines, suggesting a role of tau hyperphosphorylation in inducing inflammation.

TGF-β plays a critical role in the progression of fibrogenesis through intracellular signaling pathways, making it a key player in the pathology of DCM [51,52]. Specifically, the TGF-β1 isoform has been directly linked to myocardial fibrosis through activation of the TGF-β1/smad3 signaling pathway [6]. Additionally, TGF-βactivation in response to cardiac injury has been shown to promote cardiac hypertrophy, cardiomyocyte apoptosis, and EF reduction in post-infarction myocardium [53,54]. Excitingly, in that same spirit, our study demonstrates that TGF-β1 upregulation in T2DM can be attenuated through both daily and every other day treatments with LiCl, indicating a significant protective and reversing effect against cardiac hypertrophy and fibrosis. The modulation of smad3 phosphorylation, a key event downstream of TGF-β1 signaling, further underscores the therapeutic potential of LiCl in mitigating the adverse cardiac effects associated with T2DM by inhibiting the TGF-β1 pathway. Furthermore, studies have suggested a possible involvement of the TGF-β1 signaling pathway in tau pathology and tangle formation in neurons [55,56,57]. The relationship between TGF-β1 and tau protein in DCM remains to be fully elucidated, but our data support the notion that these two molecular mediators may be interconnected in the pathology of DCM. These findings provide promising insights into potential novel therapeutic strategies by targeting tau hyperphosphorylation to treat diabetes-induced cardiac injury.

Taken together, our findings propose that regulation of tau protein phosphorylation, to achieve a finely balanced level, can potentially ameliorate cardiac dysfunction in individuals with T2DM. In fact, studies have demonstrated that in the hearts of tau knockout mice, the absence of tau has been associated with an increase in systolic blood pressure and heart hypertrophy at 13 months of age, followed by a decline in left atrial contractility at 23 months, indicating an important functional role of tau in cardiovascular health that worsens with age [58]. Additionally, several studies delineate the emerging concept of a potential common pathogenesis for HF and Alzheimer’s disease (AD). Both T2DM and HF are risk factors for AD, and recent studies have shown that AD pathology can promote HF and vice versa, resulting in a reciprocal interaction that amplifies their mutual pathogenic effects [59]. Insulin resistance or insulin deficiency, known to play a major role in DCM, also impairs GSK-3β kinase activity and caused tau protein hyperphosphorylation in a transgenic mouse model with AD [60]. Our results, when combined with these observations, suggest that finely balancing tau protein phosphorylation can improve cardiac dysfunction in T2DM by regulating TGF-β. This study describes a potential therapeutic strategy for improving cardiovascular health in individuals with T2DM.

## 5. Conclusions

In conclusion, our findings shed light on the potential role of tau protein in maintaining cardiac function in T2DM rats. Our study provides new molecular and histological evidence that suggests a correlation between GSK-3β, tau protein hyperphosphorylation, and the TGF-β signaling pathway in the development of DCM. Importantly, our results demonstrate that a therapeutic dose of 1 mmol/kg LiCl every other day offers similar protective effects as daily treatment, potentially offering a more tolerable treatment option with fewer side effects. These findings represent a significant step towards the development of novel therapeutic strategies for the treatment of T2DM with less burden. Moving forward, further studies are needed to elucidate the precise molecular pathways that regulate the interplay between tau protein and TGF-β signaling in DCM.

Overall, our study highlights the importance of tau protein hyperphosphorylation as a potential contributor of cardiac function and underlines the importance of future studies to further assess its importance as a therapeutic target for the treatment of DCM. With further research and development, the insights gained from this study may lead to the development of safe and effective treatments for this devastating disease, offering hope to the millions of individuals affected by DCM worldwide.

## Figures and Tables

**Table 1:** Functional parameters.

Parameter	Control	T2DM	T2DM Treated with LiCl Daily	T2DM Treated with LiCl Every Other Day
Body weight (g)	350.10 ± 62.07	456.40 ± 20.59 *	347.20 ± 24.82 ^#^	491.3 ± 4.93 *^
Blood glucose (mg/dL)	114.58 ± 5.81	355.45 ± 22.69 *	302.04 ± 40.03 *	347.93 ± 34.20 *
Insulin (pmol/L)	120 ± 16	207 ± 21 *	167 ± 18 *^#^	158 ± 16 *^#^
FFA (mmol/L)	1.7 ± 0.05	5.2 ± 0.12 *	3.4 ± 0.09 *^#^	3.7 ± 0.07 *^#^
TG (mmol/L)	1.2 ± 0.02	9.9 ± 0.36 *	5.3 ± 0.44 *^#^	4.9 ± 0.33 *^#^
Tibia length (cm)	4.60 ± 0.05	3.89 ± 0.08 *	4.62 ± 0.04 ^#^	4.48 ± 0.06 ^#^
HW/TL (g/cm)	0.38 ± 0.04	0.53 ± 0.03 *	0.37 ± 0.02 ^#^	0.40 ± 0.02 ^#^

Body weight (g) and blood glucose (mg/dL) were measured weekly across the study duration of 22 weeks. Insulin (pmol/L), FFA (mmol/L), and TG (mmol/L) were measured after the sacrifice. Heart weight (g) and tibia length (cm) were measured on the sacrifice day of the rats. Values are means ± SEM; n = 5/group. * *p* < 0.05 vs. control rats; # *p* < 0.05 vs. T2DM rats; ^ *p* < 0.05 vs. T2DM treated with LiCl daily.

**Table 2:** Hemodynamic and cardiac parameters.

Parameter	Control	T2DM	T2DM Treated with LiCl Daily	T2DM Treated with LiCl Every Other Day
LV Structure				
LVM (g)	1.73 ± 0.32	1.81 ± 0.10	1.51 ± 0.07	1.66 ± 0.10
LVM/TL (g/cm)	0.32 ± 0.04	0.46 ± 0.02 *	0.33 ± 0.02 ^#^	0.38 ± 0.01 ^#^
FS %	49.11 ± 0.68	31.84 ± 2.08 *	44.24 ± 3.36 ^#^	41.67 ± 3.16 ^#^
EDD (mm)	7.12 ± 0.70	7.91 ± 0.67	7.27 ± 0.25	7.69 ± 0.19
ESD (mm)	3.64 ± 0.38	5.39 ± 0.26 *	4.08 ± 0.22 ^#^	4.58 ± 0.3 *
LV Function				
EF %	84.60 ± 0.51	64.68 ± 3.28 *	79.56 ± 3.54 ^#^	76.20 ± 3.60 ^#^
EDV (Teich) (mL)	0.93 ± 0.23	1.19 ± 0.19	0.91 ± 0.08	1.02 ± 0.07
ESV (Teich) (mL)	0.15 ± 0.03	0.40 ± 0.05 *	0.18 ± 0.03 ^#^	0.24 ± 0.04 ^#^
Representative 2D Echo	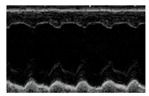	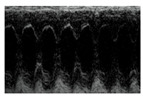	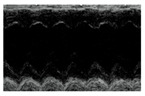	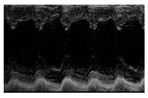

LV functional parameters in control rats, rats with T2DM, and T2DM rats treated with LiCl daily or every other day. LVM: LV mass; FS: fractional shortening; EDD: end-diastolic diameter; ESD: end-systolic diameter; EF: ejection fraction; EDV: end-diastolic volume; ESV: end-systolic volume; Teich: Teichholz formula (Vol = 7D^3/(2.4 + D)). The values are presented as means ± SEM with n = 5/group. Statistical significance is denoted by * *p* < 0.05 vs. control rats, # *p* < 0.05 vs. T2DM rats.

## Data Availability

The data presented in this study are available upon request from the corresponding author upon reasonable request.

## Supplementary Materials

The following supporting information can be downloaded at: https://www.mdpi.com/article/10.3390/pharmaceutics16070955/s1.
