# Supplementary material for: Uncovering the Therapeutic Potential of Lithium Chloride in Type 2 Diabetic Cardiomyopathy: Targeting Tau Hyperphosphorylation and TGF-β Signaling via GSK-3β Inhibition
Source: Pharmaceutics. 2024 Jul 19;16(7):955. doi: 10.3390/pharmaceutics16070955 (PMC11279906; doi:10.3390/pharmaceutics16070955)
Supplement: Supplementary file 1 [file pharmaceutics-16-00955-s001.zip › pharmaceutics-2550908-supplementary.pdf]

Supplementary Material

Figure S1

Phospho-Tau S214

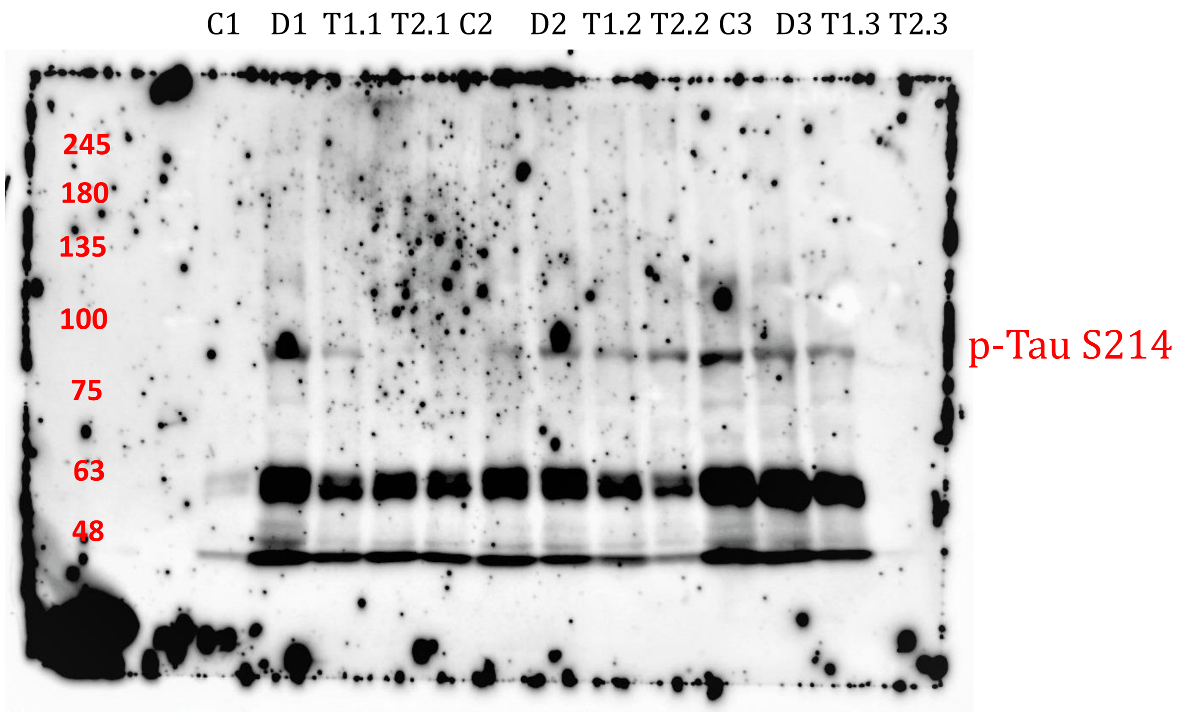

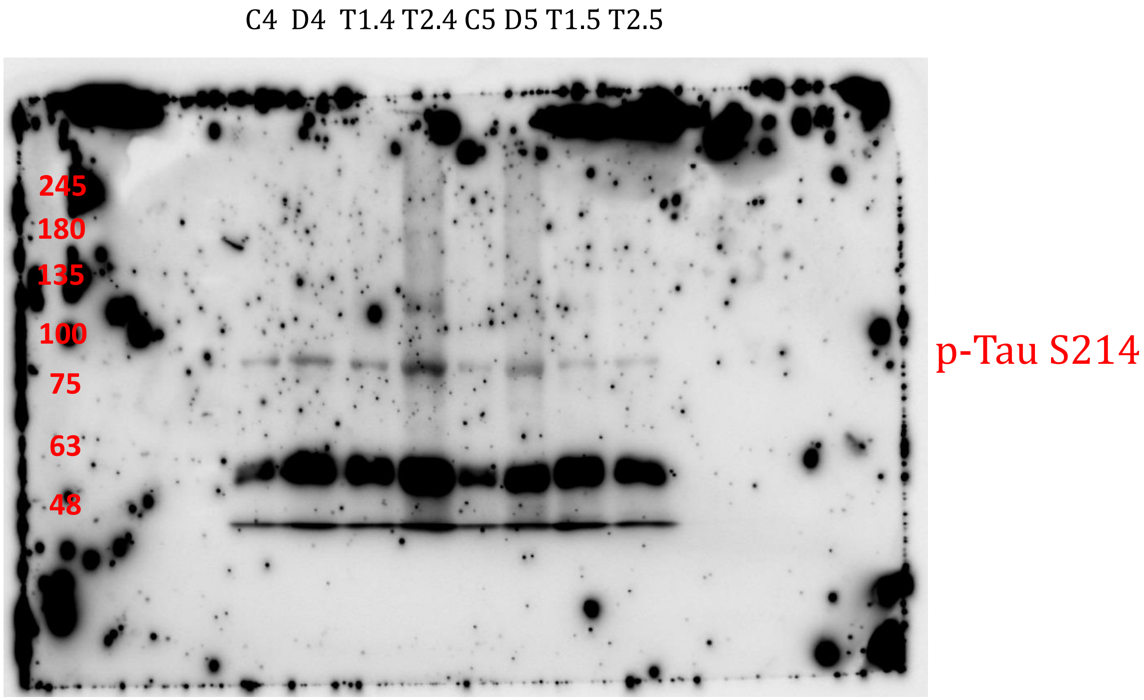

Gels 8%
Phospho-Tau S214 Antibody #10891
Anti-Rabbit 1:250
MW: 79

Figure S1

HSC-70(for p-Tau S214)

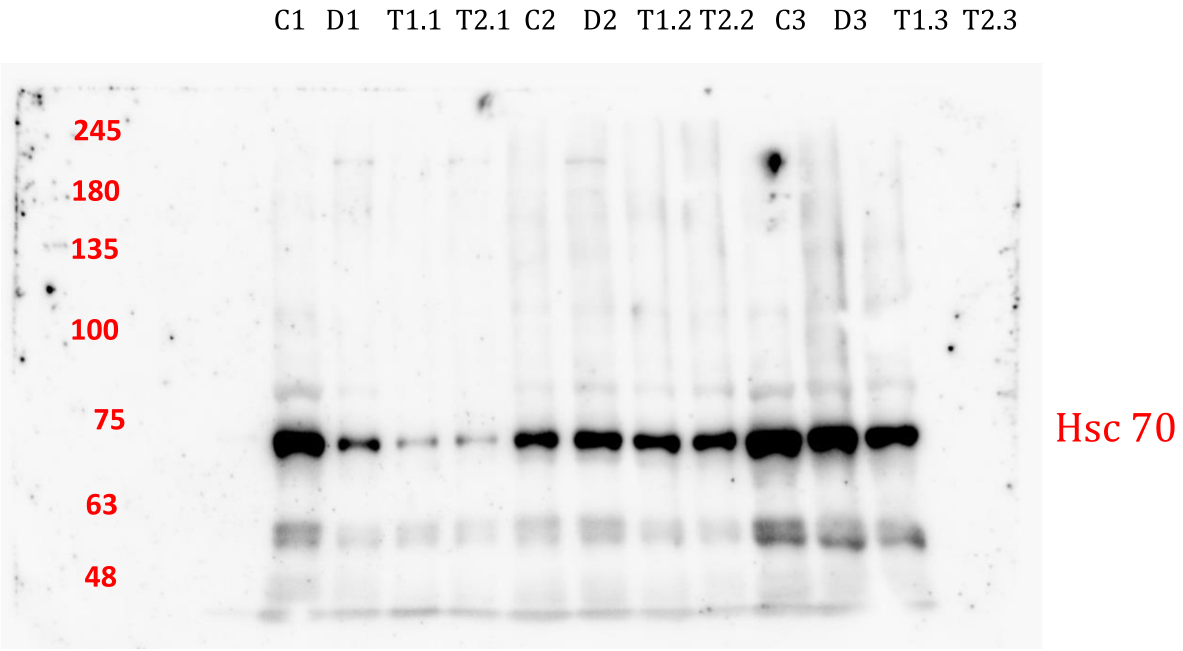

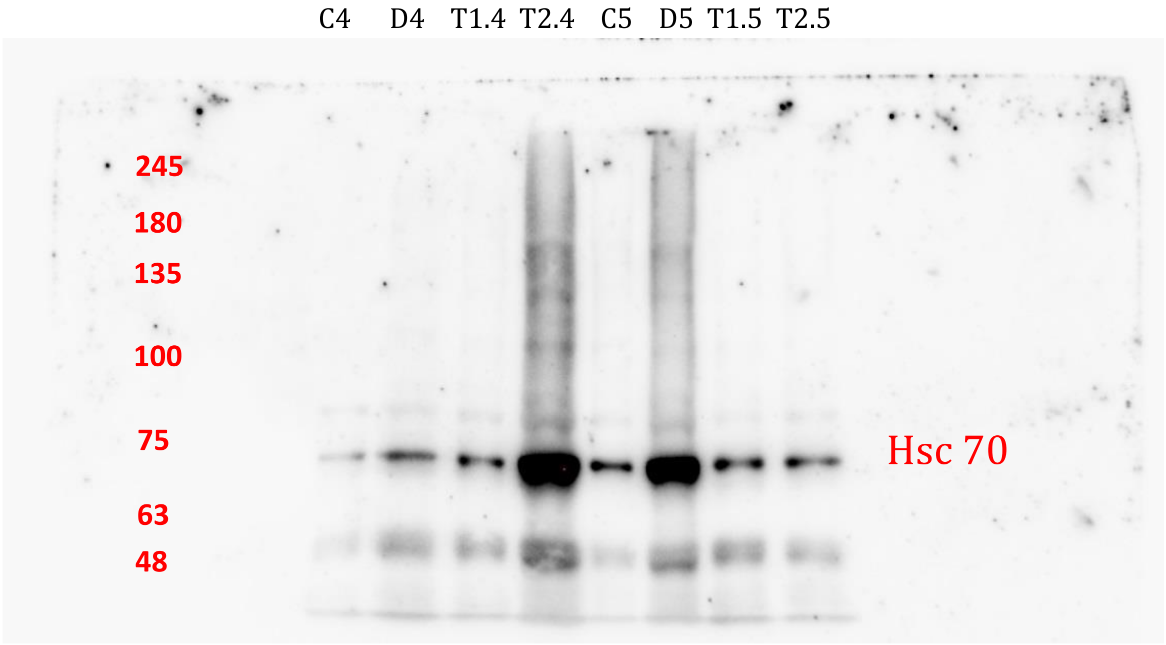

Gels 8%
Hsc 70 (B-6) Antibody #7298
Anti-Mouse 1:500
MW: 70

Figure S1

Total Tau (for Tau S214)

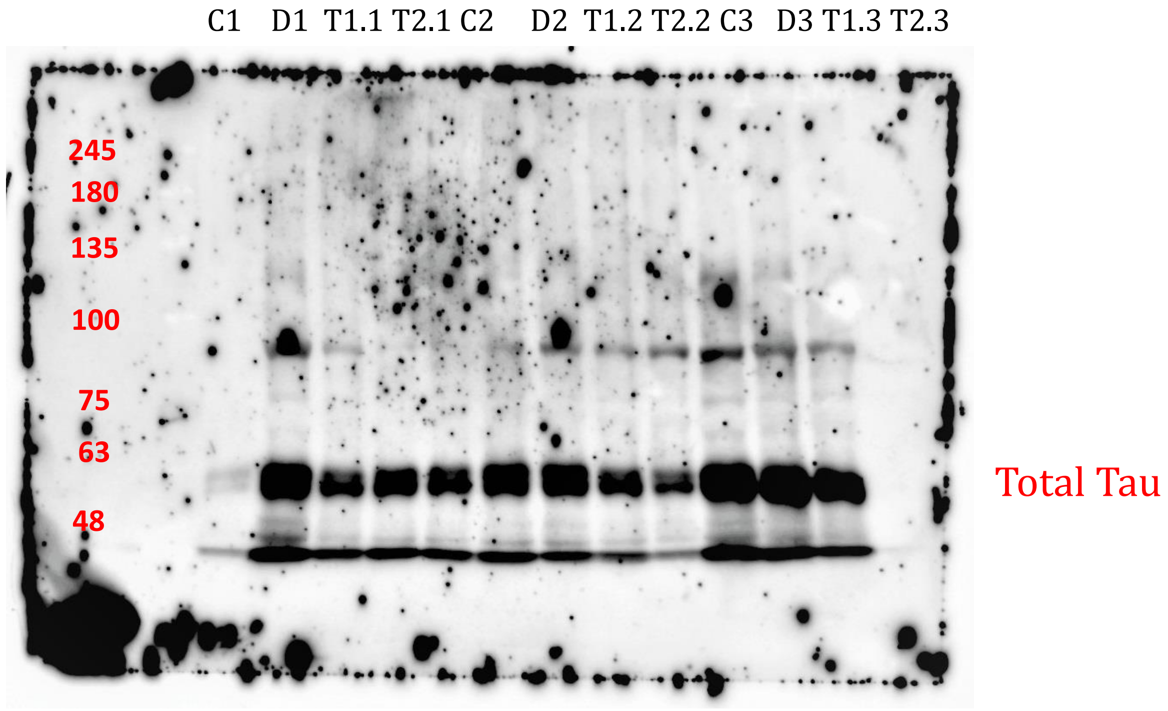

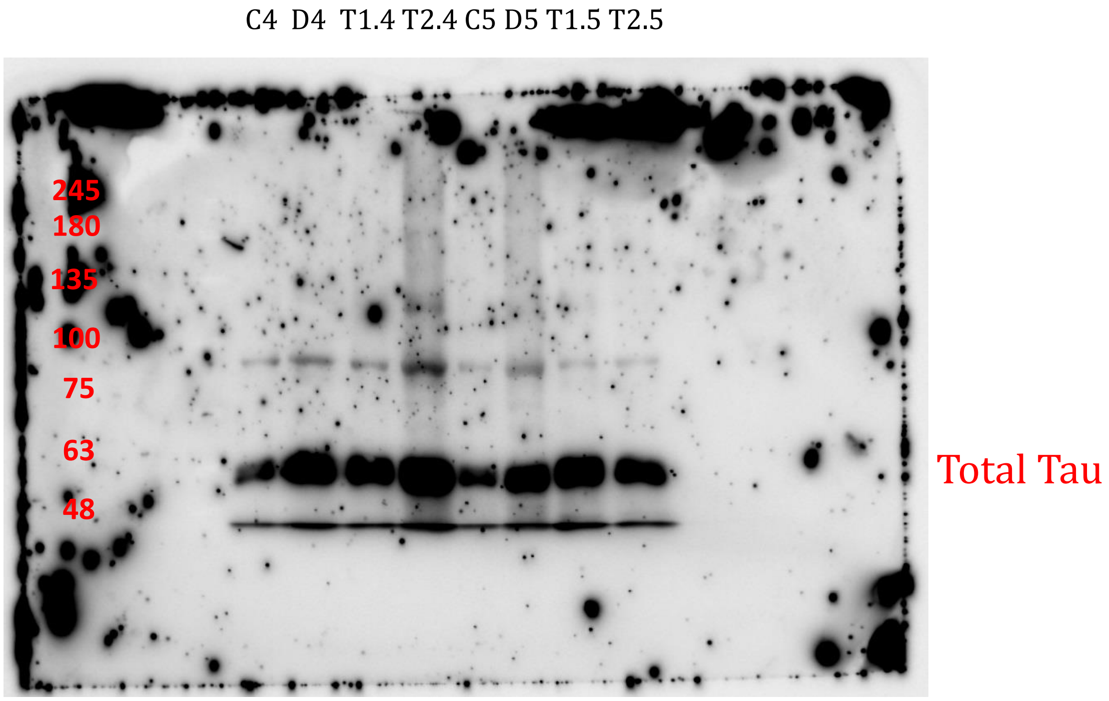

Gels 8%
Total Tau Antibody #0157R
Anti-Rabbit 1:300
MW: 50

Figure S1

Phospho-GSK-3β (Ser9)

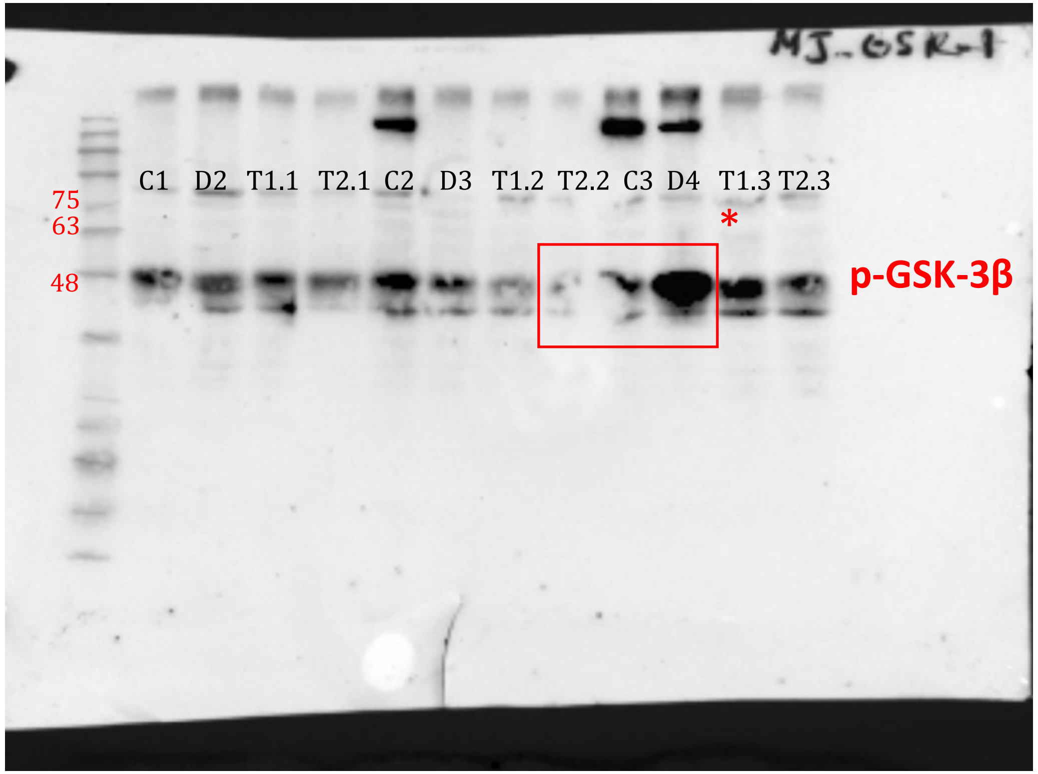

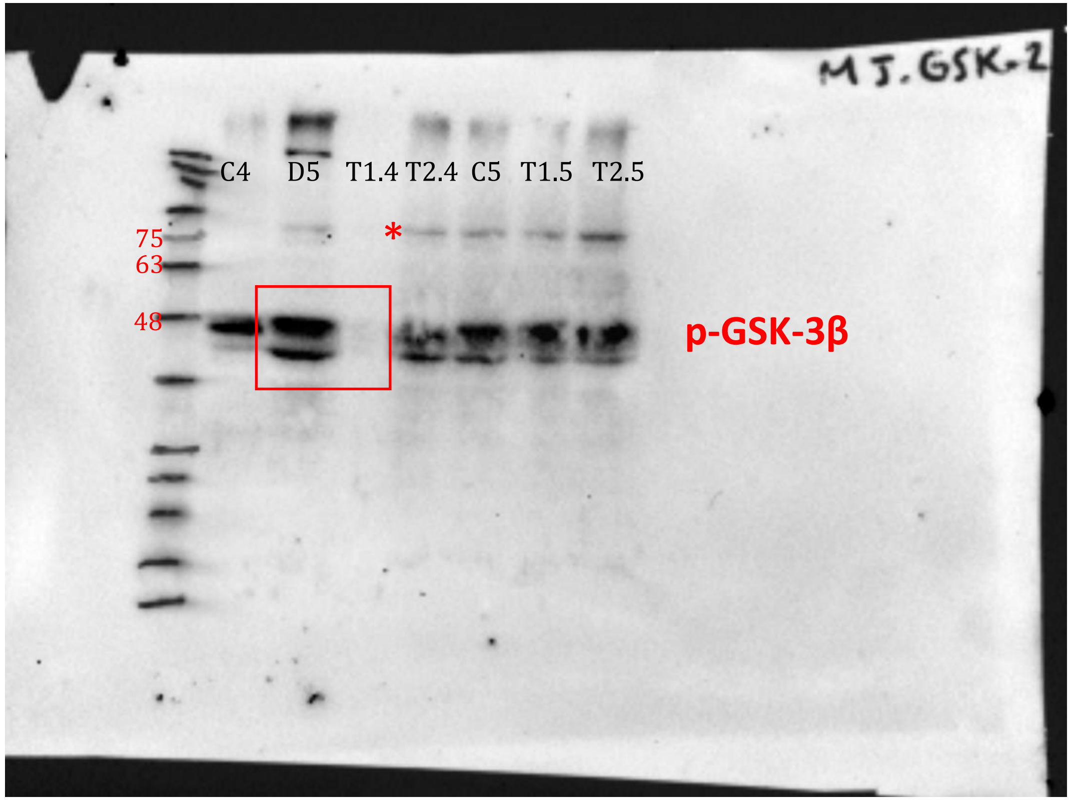

Gels 12%
Phospho-GSK-3β (Ser9) Antibody #9336
Anti-Rabbit 1:10 000
MW:46

* Samples were repeated below

Figure S1

Phospho-GSK-3β (Ser9)

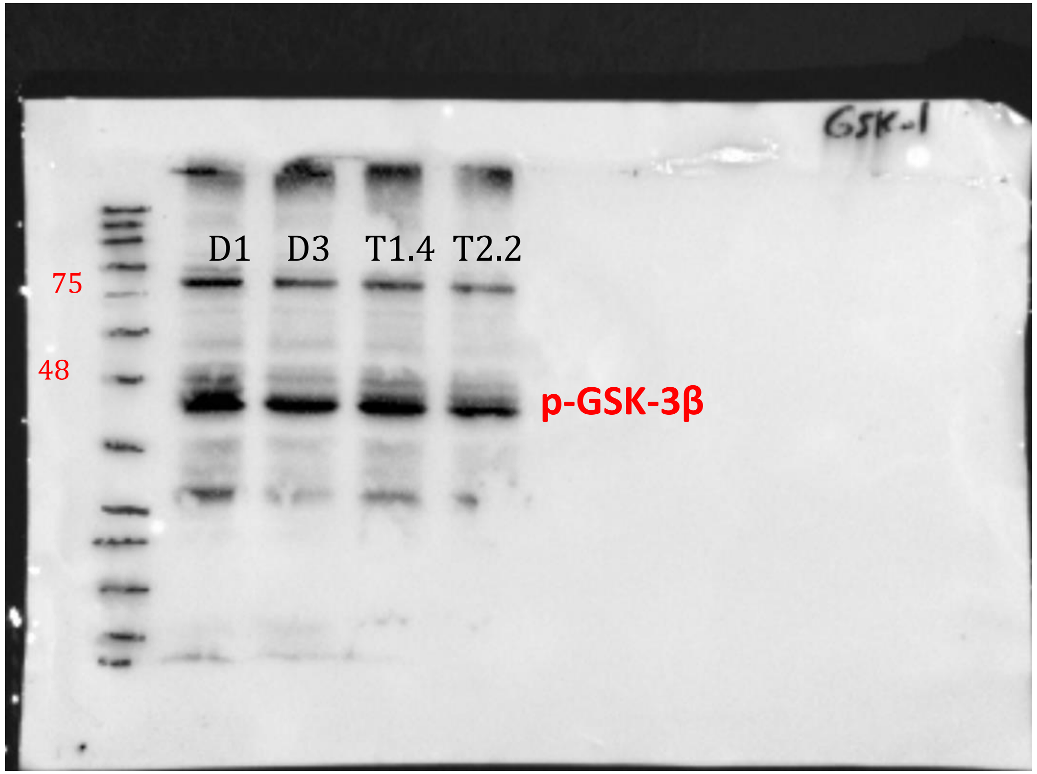

Gels 12%
Phospho-GSK-3β (Ser9) Antibody #9336
Anti-Rabbit 1:10 000
MW:46

HSC-70

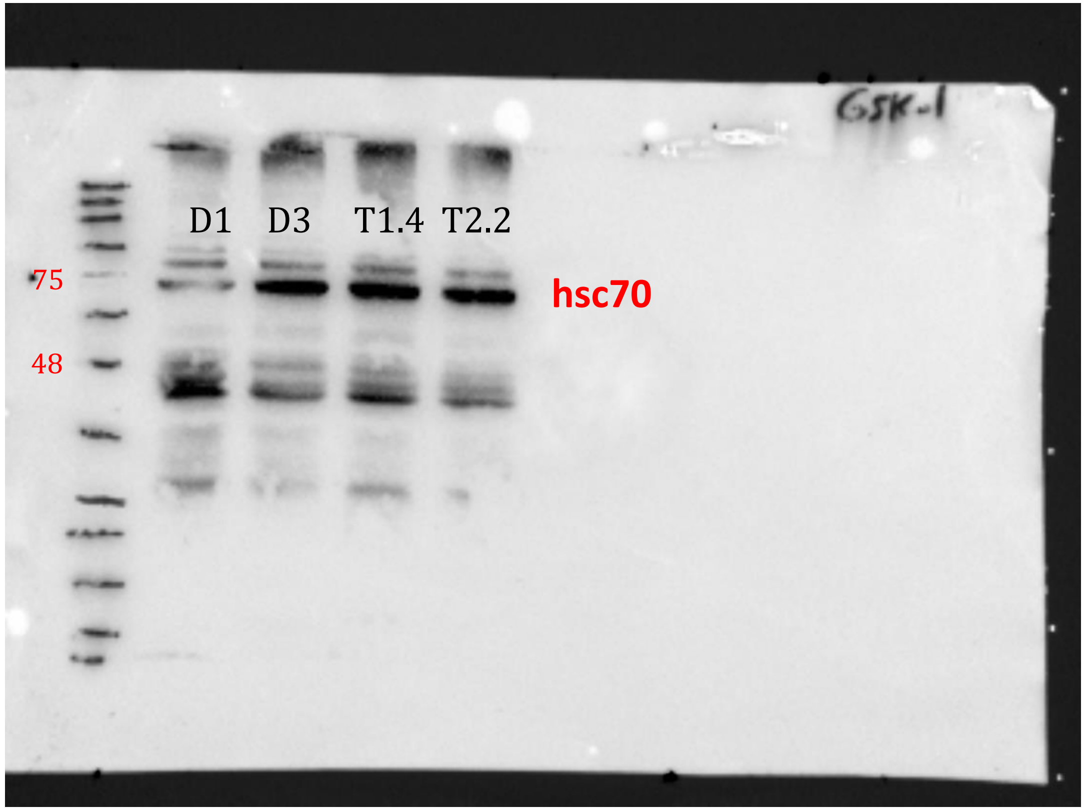

Gels 12%
HSC-70 Santa cruz ((B-6): sc-7298) 1:1000
Anti-mouse 1:20 000
MW:70

Figure S1

GSK-3β

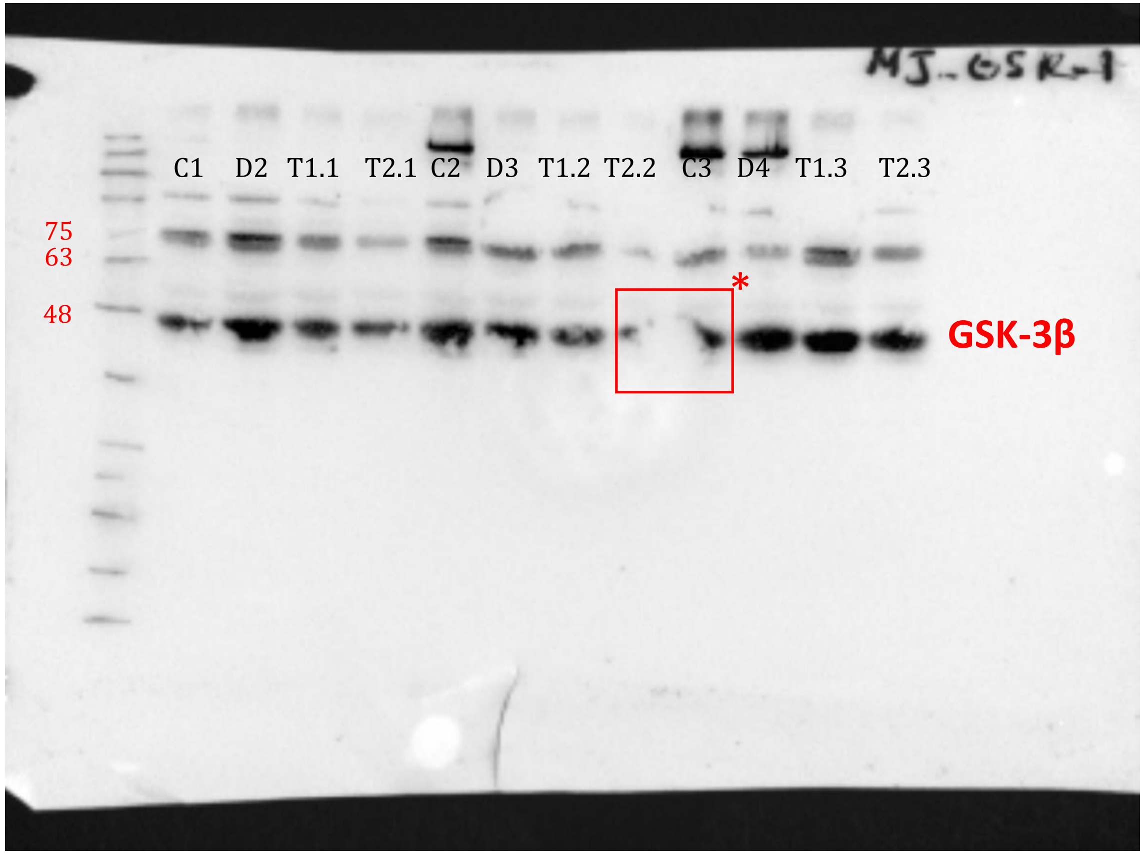

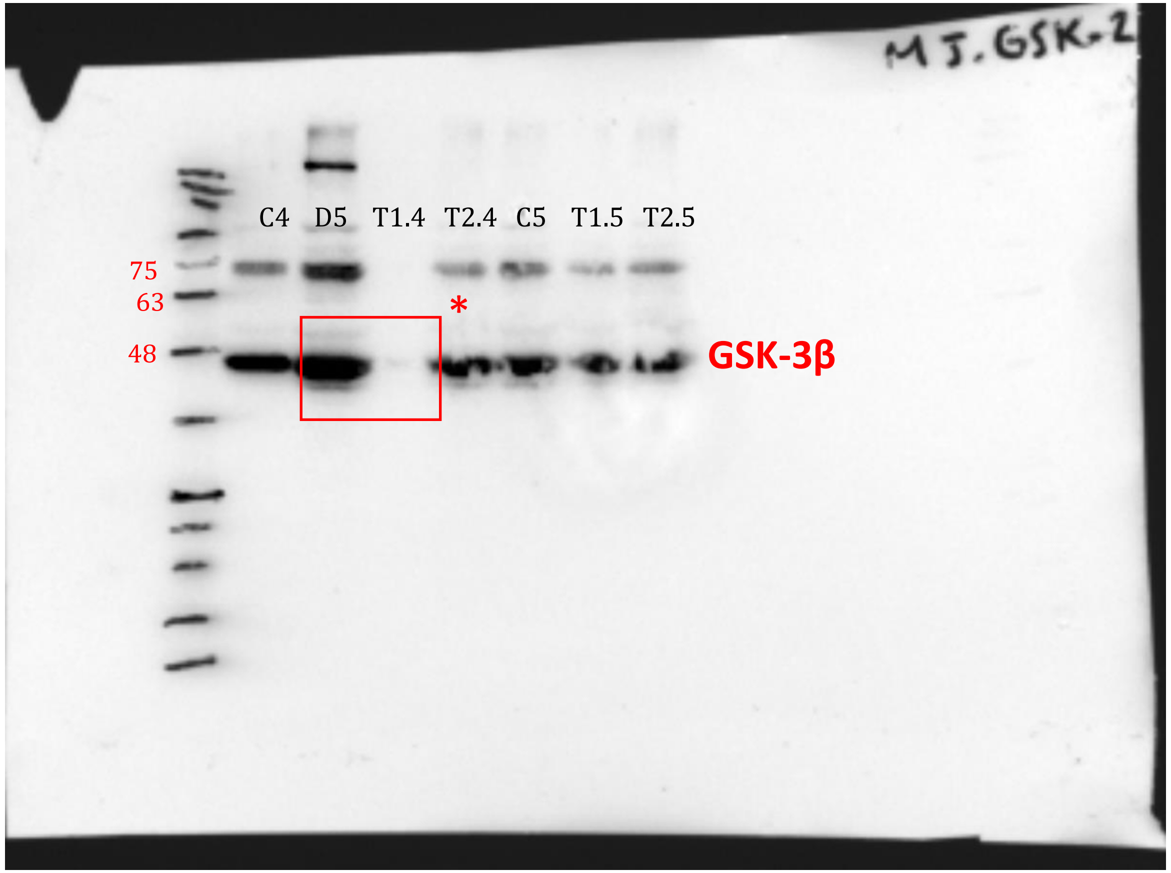

Gels 12%
GSK-3beta Antibody #9332
Anti-Rabbit 1:10 000
MW:46

* Samples were repeated

Figure S1

GSK-3beta

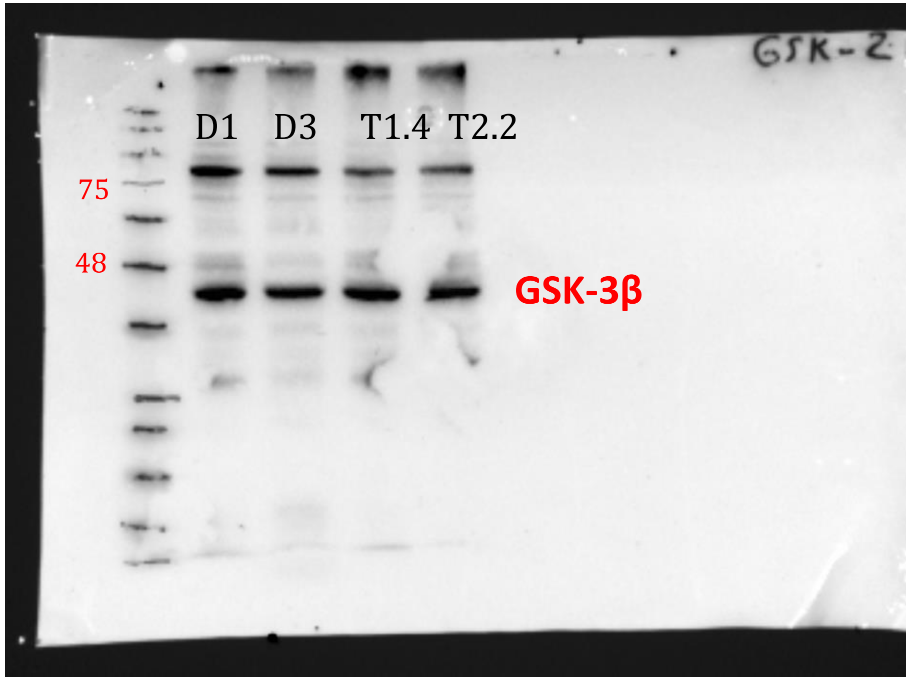

Gels 12%
GSK-3beta Antibody #9332
Anti-Rabbit 1:10 000
MW:46

HSC-70

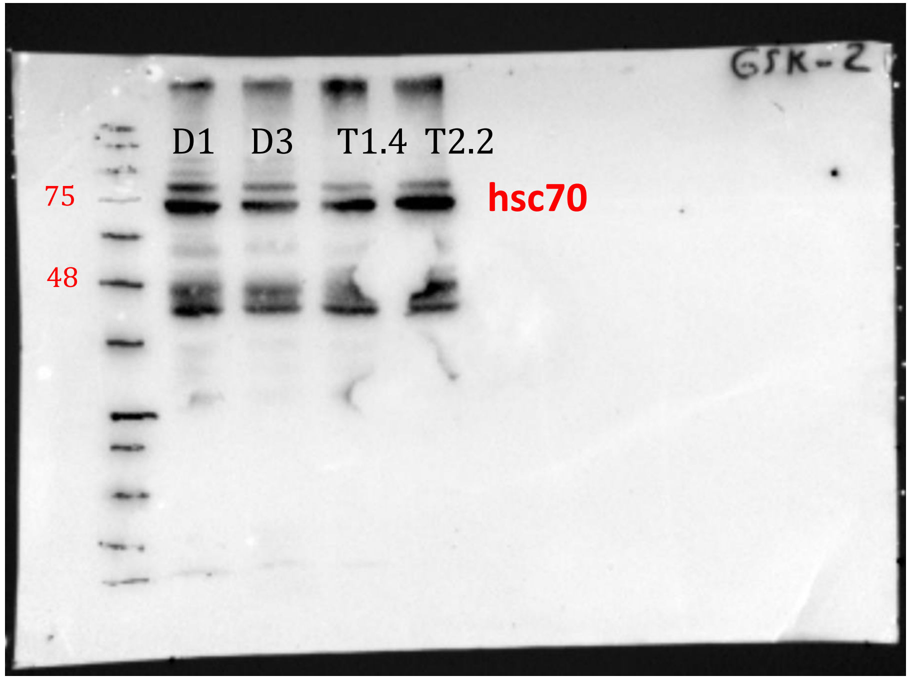

Gels 12%
HSC-70 Santa cruz ((B-6): sc-7298)
1:1000
Anti-mouse 1:20 000
MW:70

Figure S1

HSC-70 (for GSK-3β)

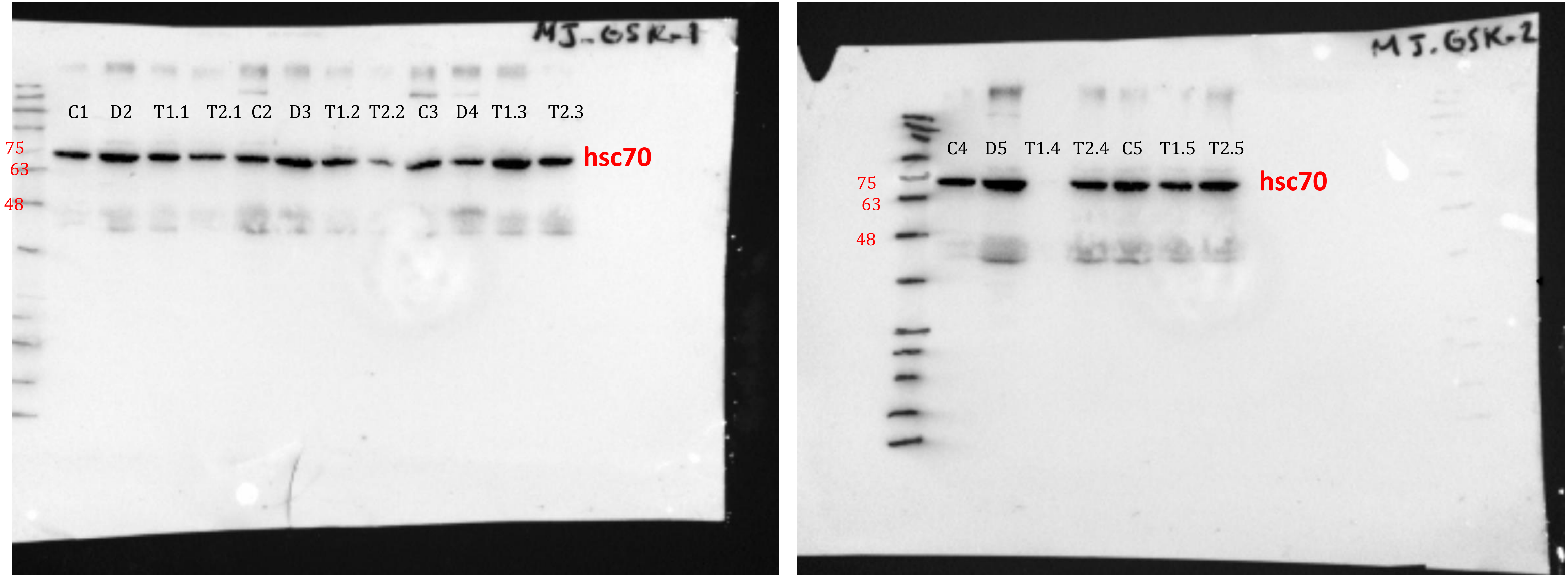

Gels 12%
HSC-70 Santa cruz ((B-6): sc-7298) 1:1000
Anti-mouse 1:20 000
MW:70

Figure S2

α-SMA

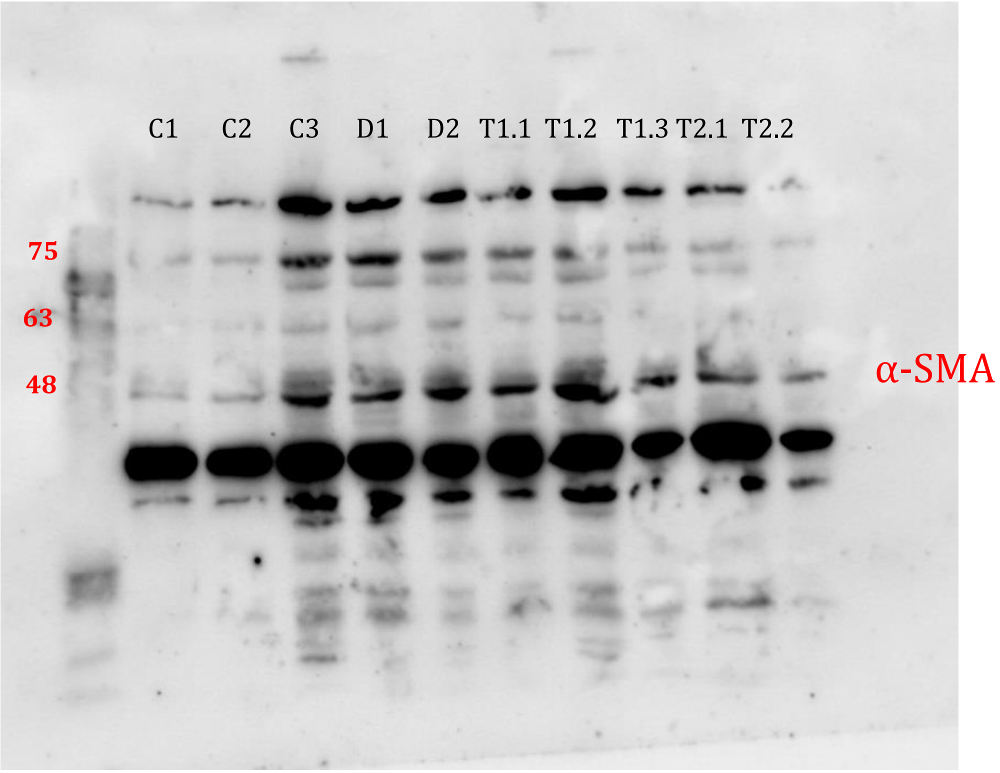

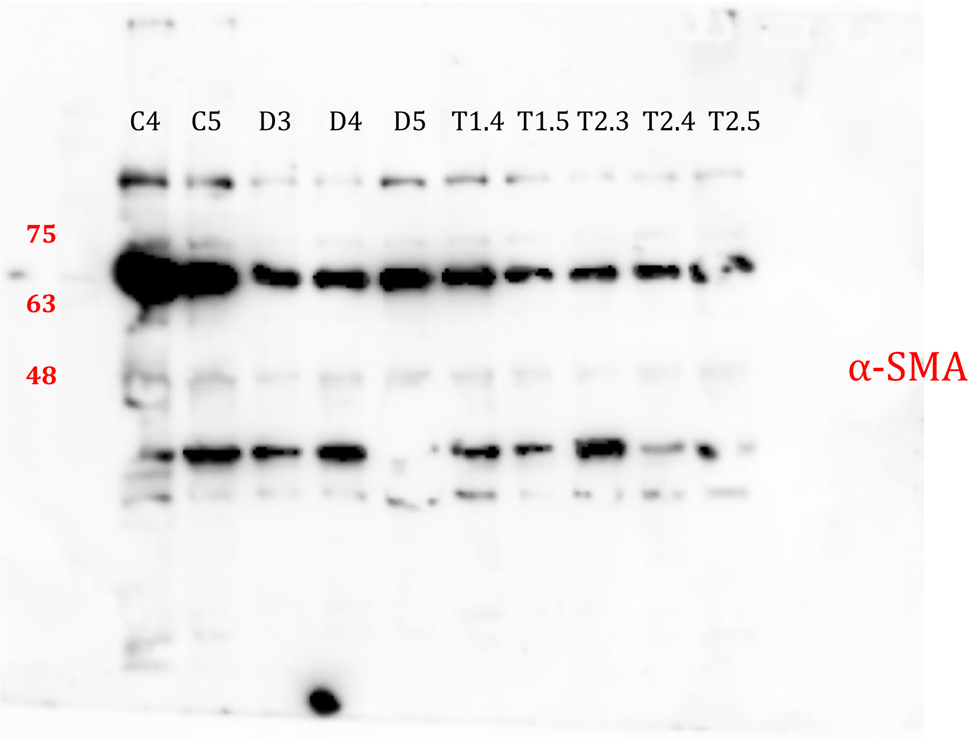

Gels 12%
α-SMA Antibody #5694
Anti-Rabbit 1:200
MW: 42

Figure S2

HSC-70 (for α sma)

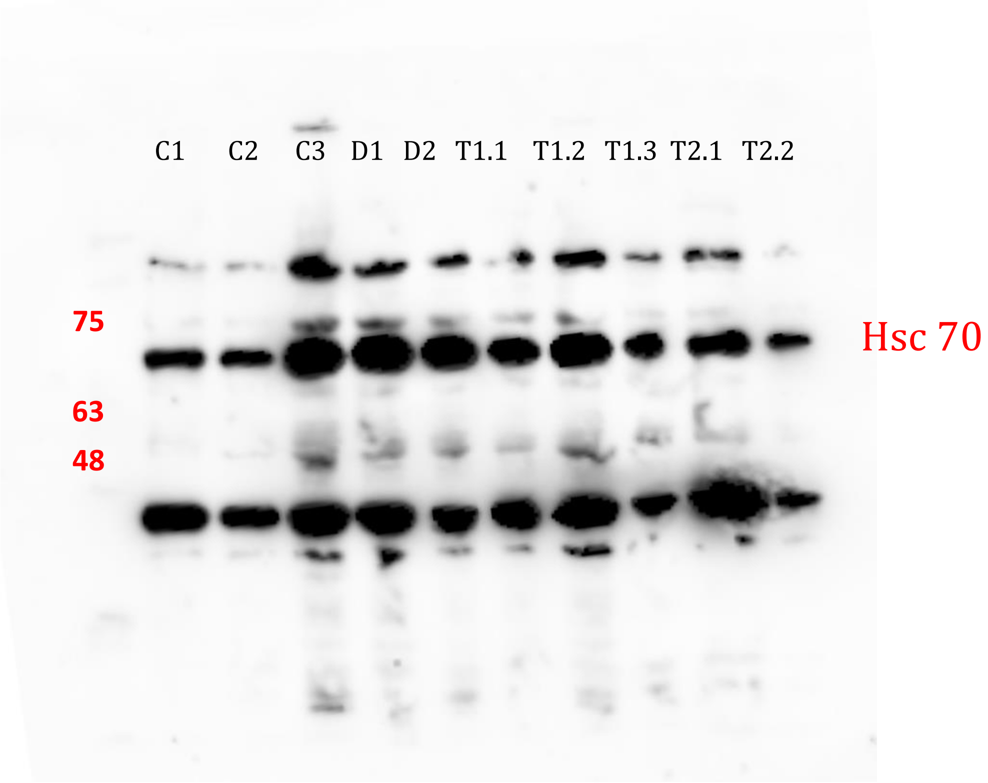

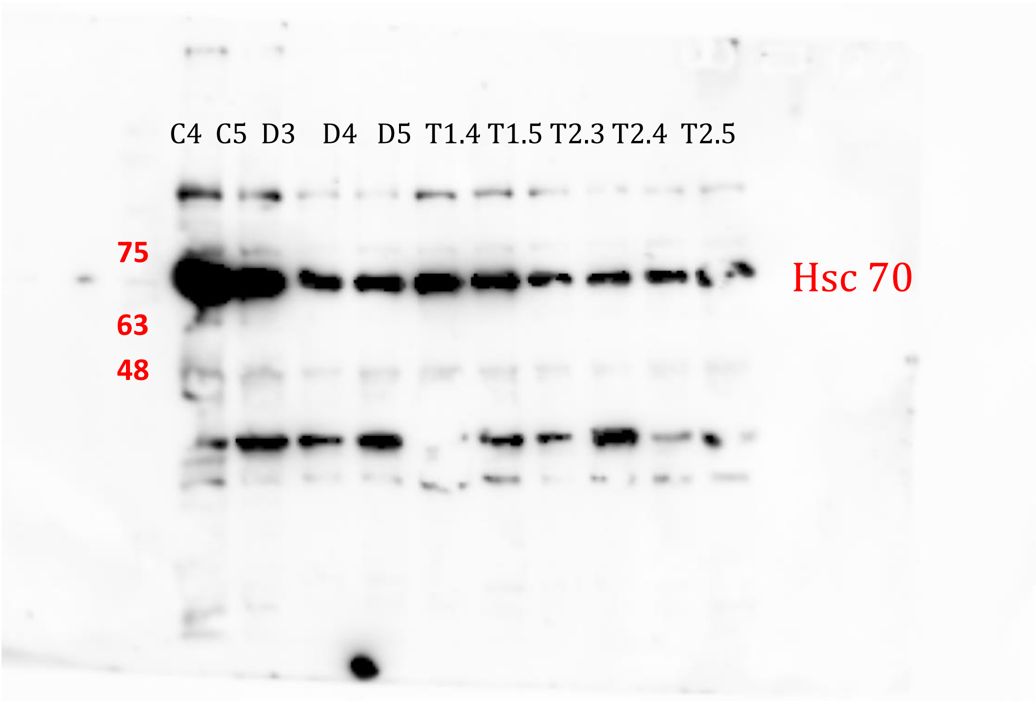

Gels 12%
Hsc 70 Antibody #7298
Anti-Mouse 1:500
MW: 70

Figure S2

MHC-alpha

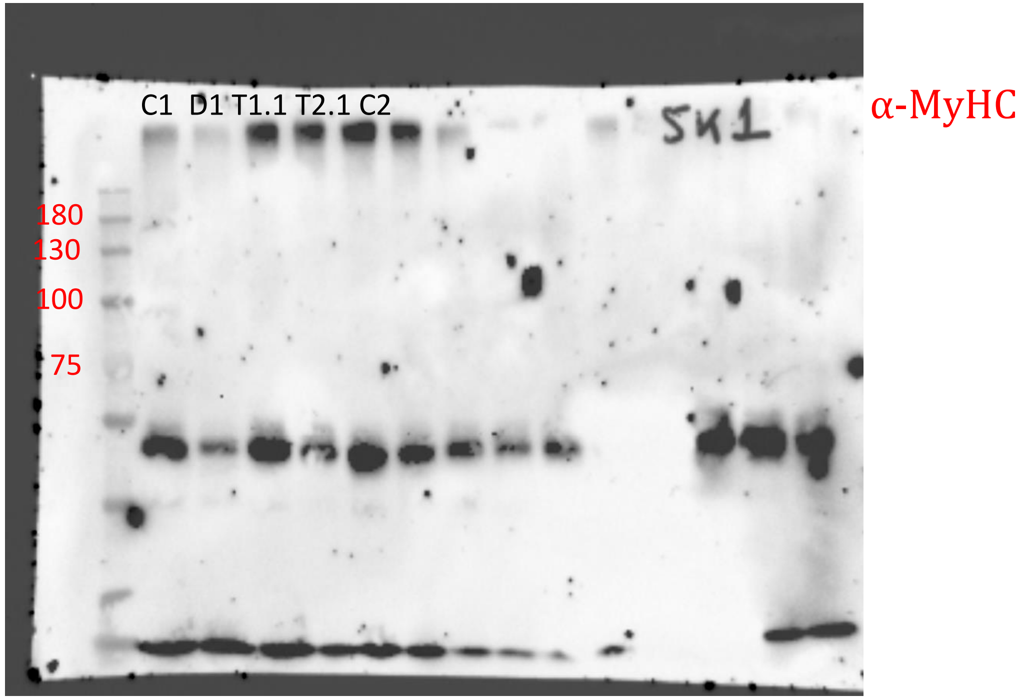

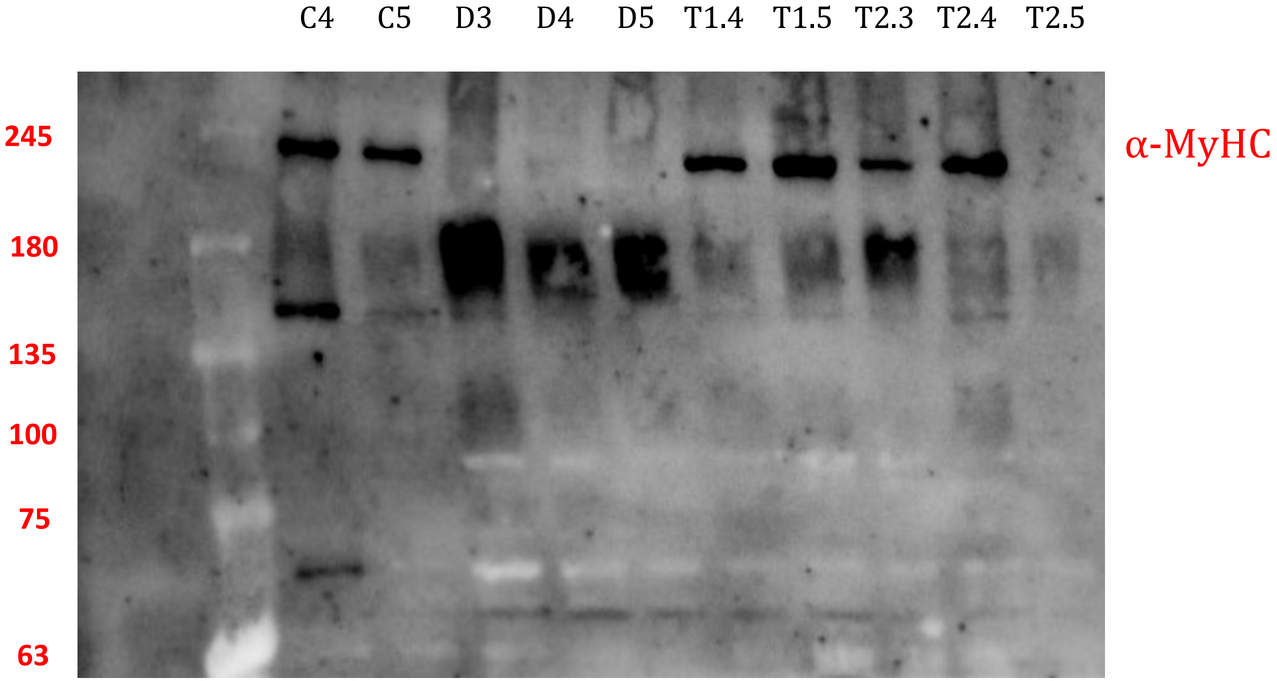

Gels 8%
MYH6(K-13) Antibody #168676
Anti-Goat 1:500
MW: 224

Figure S2

HSC-70(for MHC-alpha)

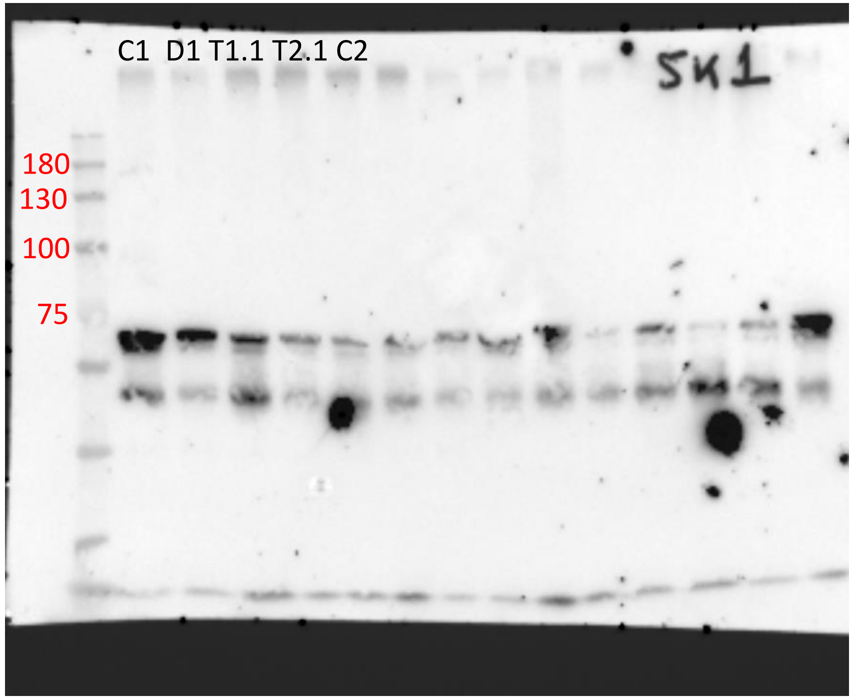

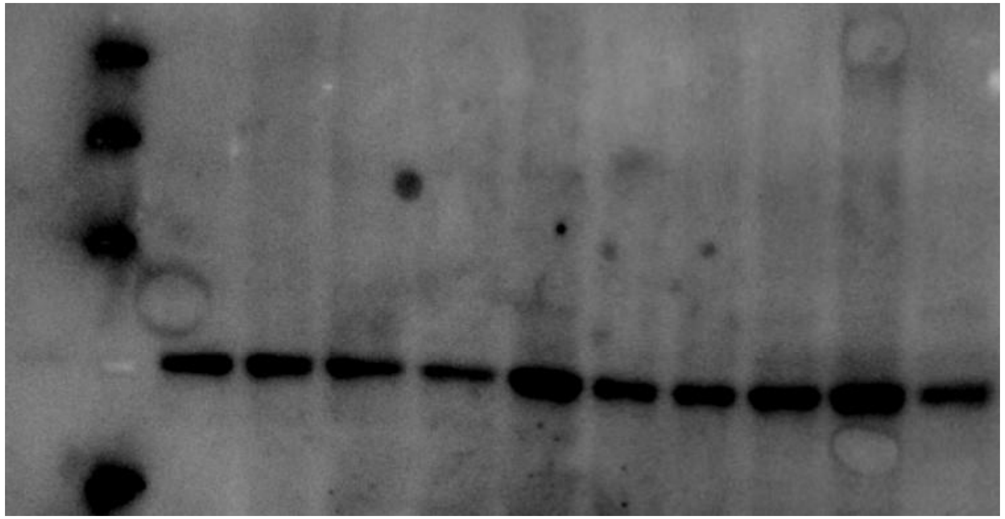

Gels 8%
Hsc 70 (B-6) Antibody #7298
Anti-Mouse 1:500
MW: 70

Figure S3

Phospho-smad (Ser423/425)

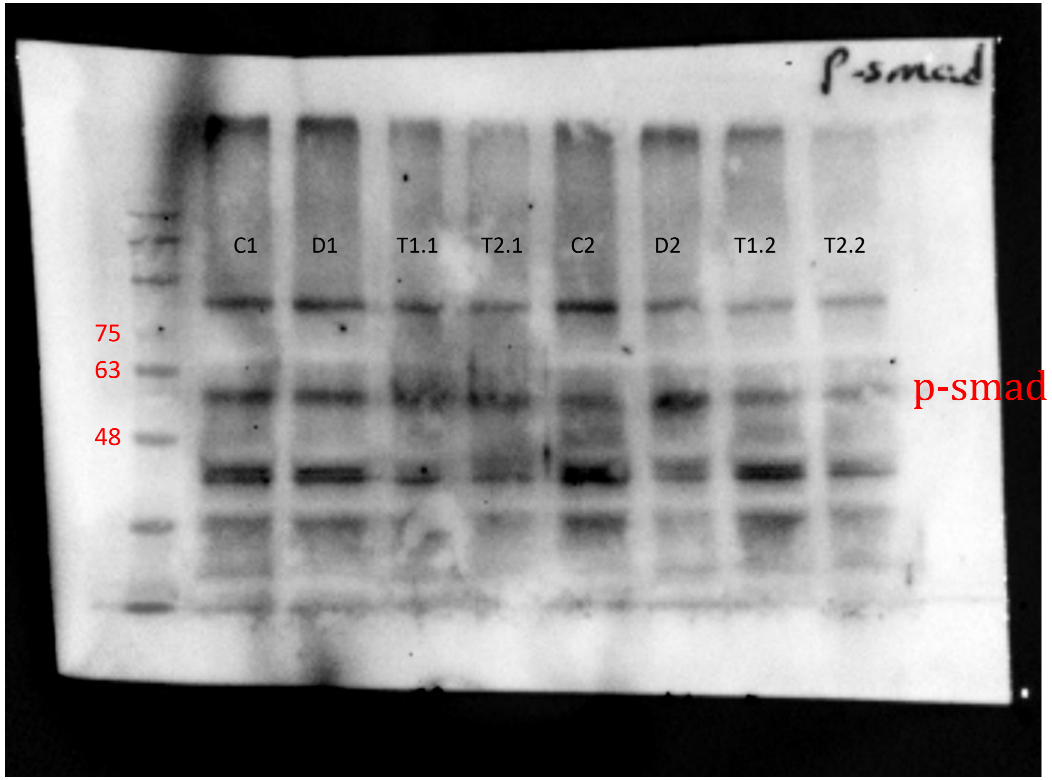

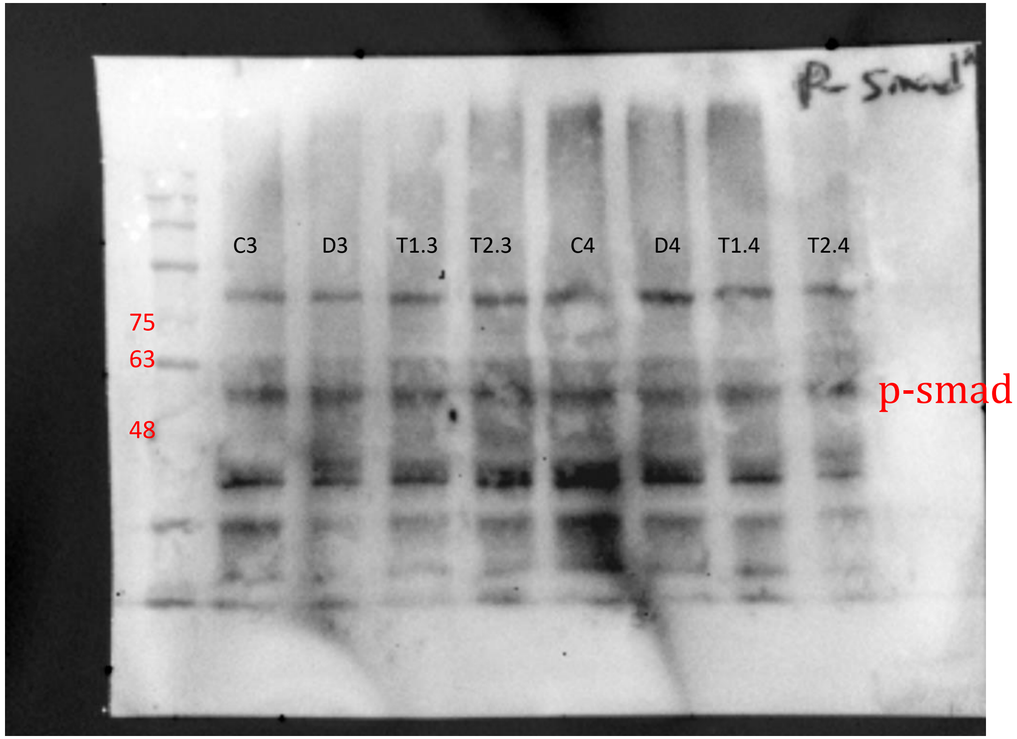

Gels 12%

Phospho-SMAD3 (Ser423/425) (C25A9) Rabbit mAb #9520

Anti-rabbit :20 000

MW:52

Figure S3

HSC-70 (for p-smad (Ser423/425))

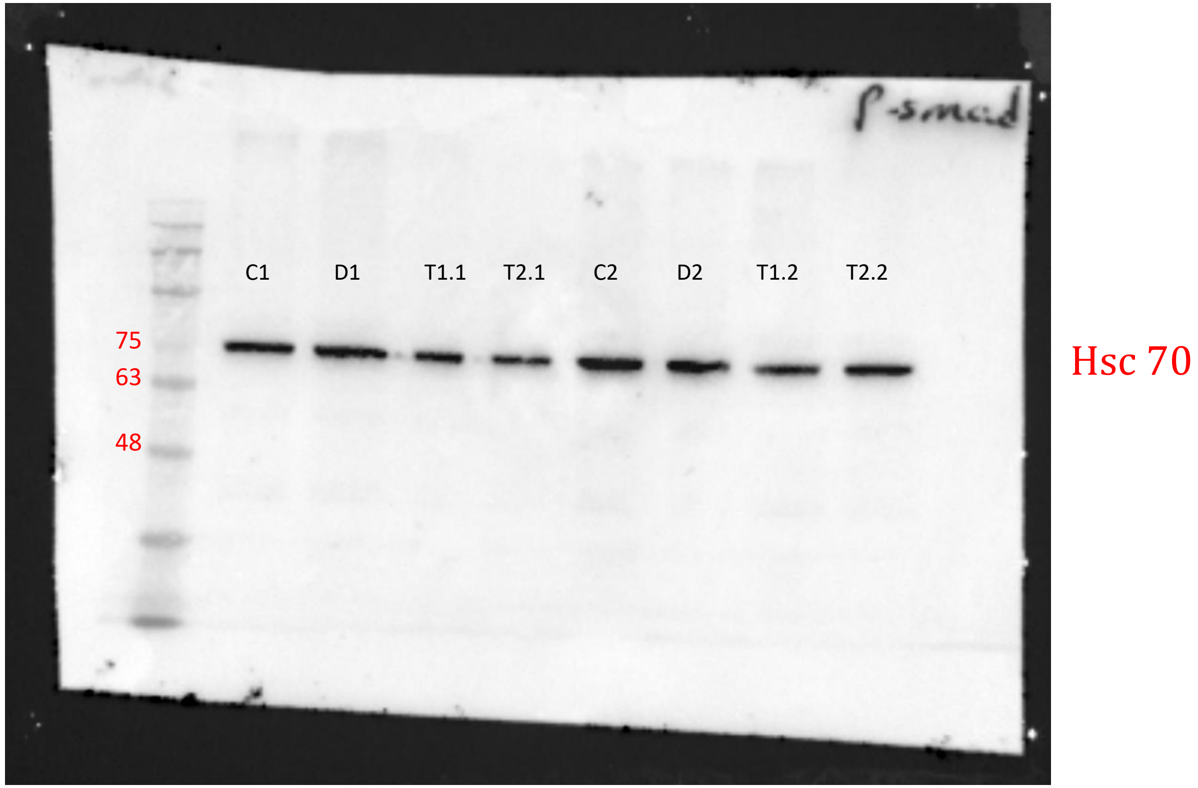

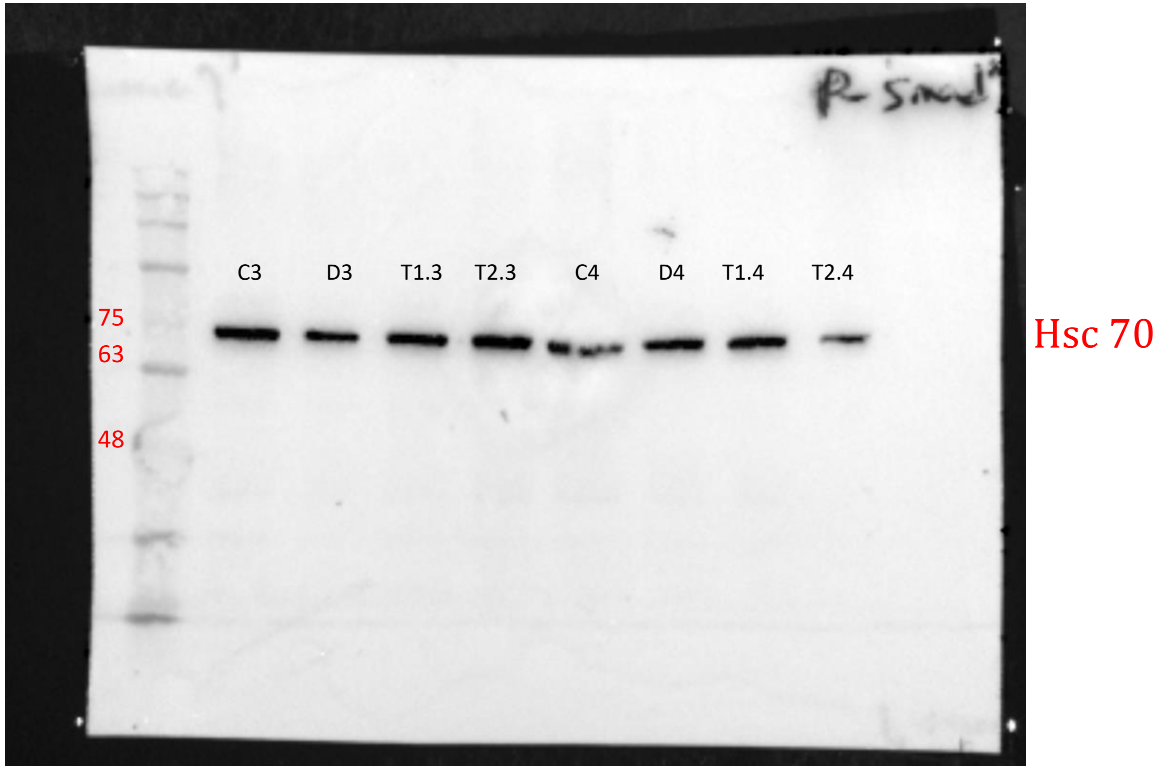

- Gels 12%
- HSC-70 Santa cruz ((B-6): sc-7298) 1:1000
- Anti-mouse 1:20 000
- MW:70

Figure S3

smad3

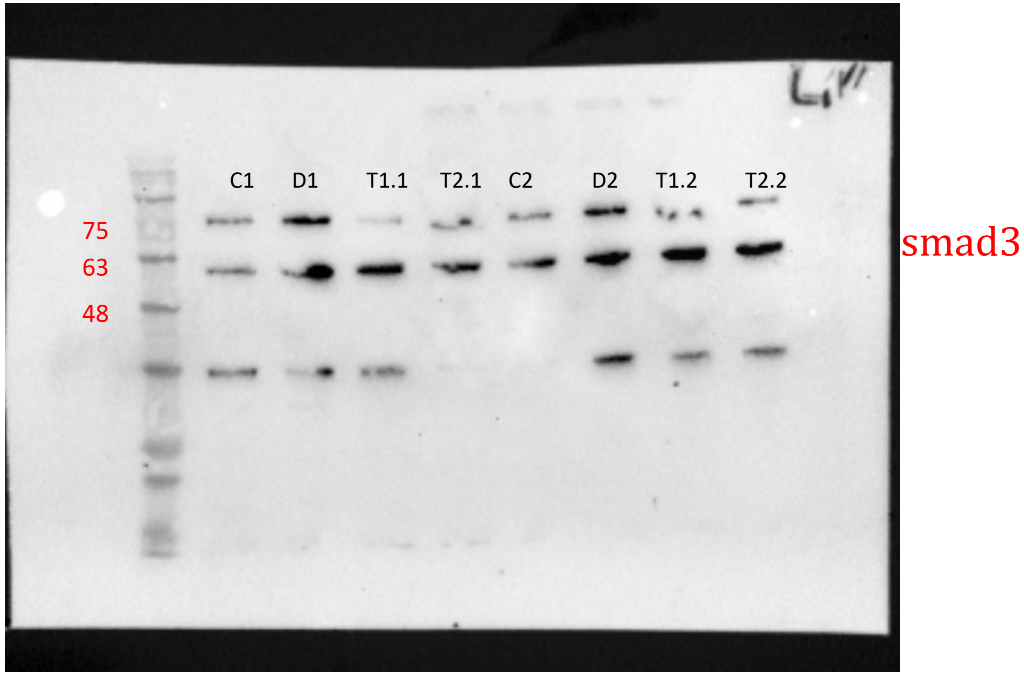

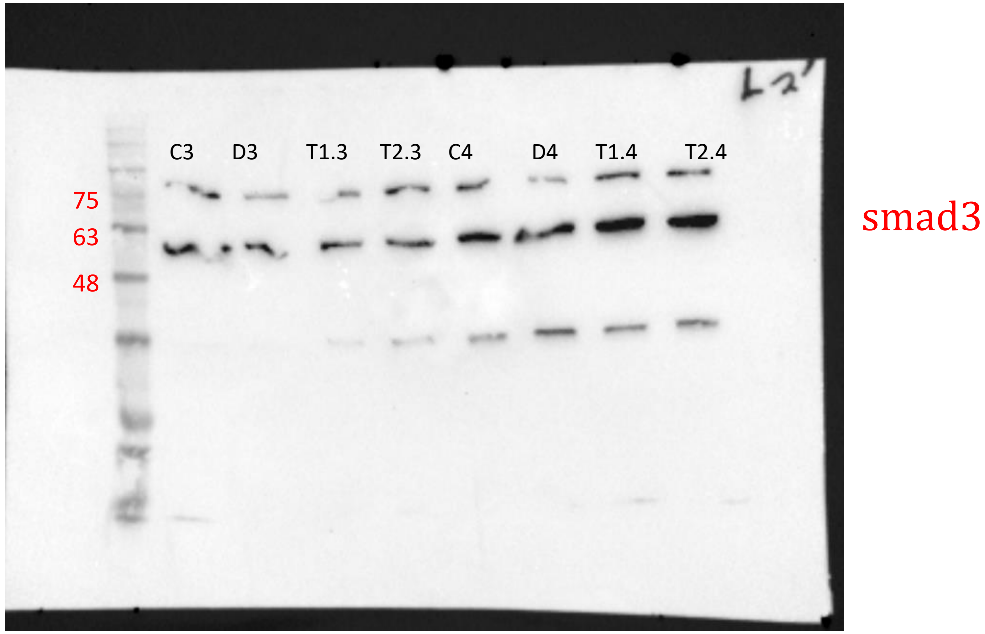

Gels 15%

SMAD3 (C67H9) Rabbit mAb #9523

Rabbit mAb #9520Anti-rabbit :20 000

MW:52

Figure S3

HSC-70 (for smad)

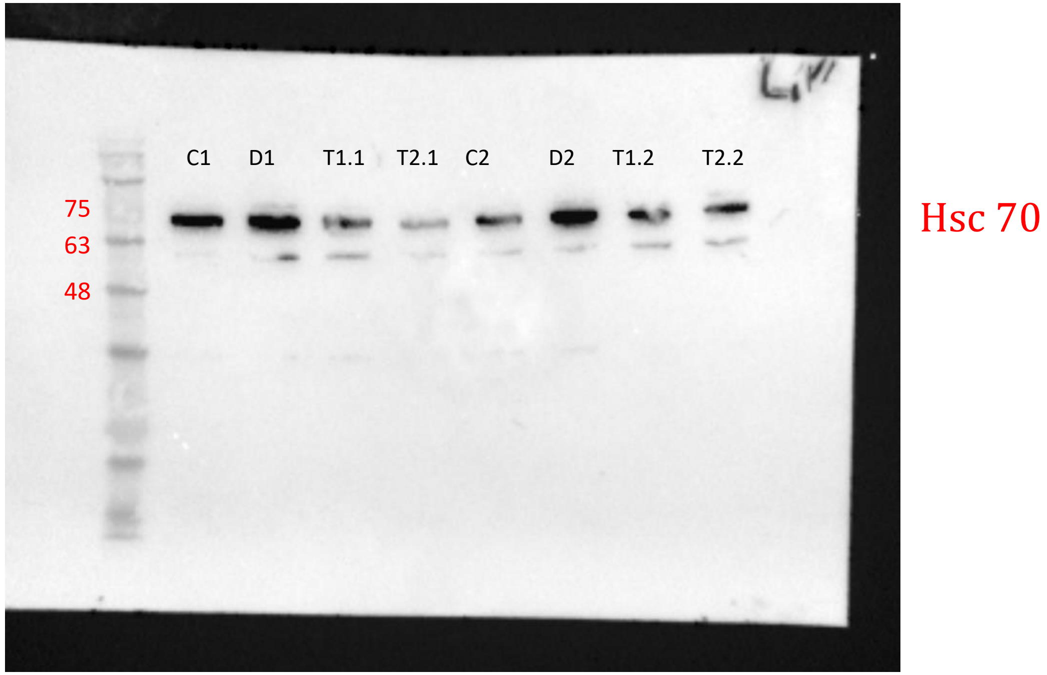

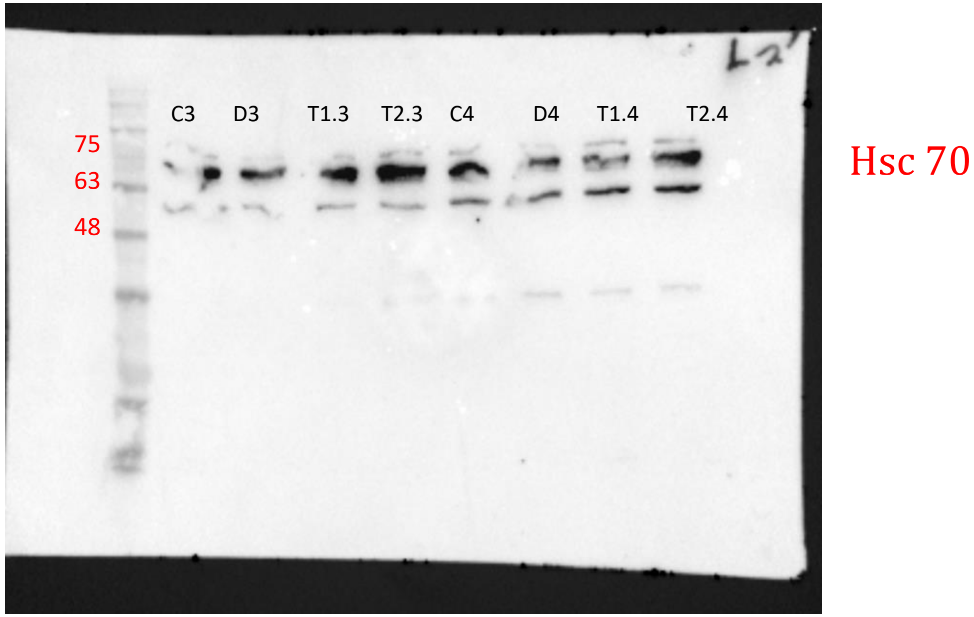

- Gels 15%
- HSC-70 Santa cruz ((B-6): sc-7298) 1:1000
- Anti-mouse 1:20 000
- MW:70

Table S1. Densitometry ratio

Sample	%(Ptau/HSC)/(Tau/HSC)	%P-GS3K/GS3K*	%sma/hsc	% MYhc/HSC	%(Psmad/HSC)/(smad/HSC)
C1	100	100	100	100	100
C2	100	100	100	100	100
C3	100	100	100		100
C4	100	100	100	100	100
C5	100	100	100	100	
T2DM 1	200.702	*340.7039**	157.8845	10.454	137.6807
T2DM 2	*74.11729**	50.76554	200.7756		170.0468
T2DM 3	190.6654	56.1155	199.8351	19.188	233.1775
T2DM 4	*52.60956**	*102.1684**	235.8895	12.102	53.68616*
T2DM 5	334.7995	67.89118	*107.1283**		
T2DM+LiCl DAILY 1	87.16124	87.72902	76.65724	105.073	38.99557
T2DM+LiCl DAILY 2	144.8465	61.43334	103.122		50.96535
T2DM+LiCl DAILY 3	*392.3688**	86.48796	69.85839		*243.5372**
T2DM+LiCl DAILY 4	69.7431	77.78506	*169.9131**	106.495	11.8305
T2DM+LiCl DAILY 5	45.68914	110.596	148.0351	56.48	
T2DM+LiCl EVERY OTHER DAY 1	33.03826	91.62428	93.2294	77.223	27.05698
T2DM+LiCl EVERY OTHER DAY 2	116.7925	79.75155	90.38271		60.65323
T2DM+LiCl EVERY OTHER DAY 3	167.0193	76.04399	*225.5634**	75.591	146.5681
T2DM+LiCl EVERY OTHER DAY 4	*405.0407**	79.63436	112.4551	63.972	*122.8239*
T2DM+LiCl EVERY OTHER DAY 5	48.70627	*160.5984**	117.3748		
